# Characterization of an Entner–Doudoroff pathway-activated *Escherichia coli*

**DOI:** 10.1186/s13068-022-02219-6

**Published:** 2022-11-09

**Authors:** Ye Eun Kim, Kyung Hyun Cho, Ina Bang, Chang Hee Kim, Young Shin Ryu, Yuchan Kim, Eun Mi Choi, Linh Khanh Nong, Donghyuk Kim, Sung Kuk Lee

**Affiliations:** 1 School of Energy and Chemical Engineering, Ulsan National Institute of Science and Technology (UNIST), Ulsan, 44919 Republic of Korea; 2 Department of Biomedical Engineering, UNIST, Ulsan, 44919 Republic of Korea

**Keywords:** *Escherichia coli*, Glucose metabolism, Entner–Doudoroff pathway, Adaptive laboratory evolution, Phosphofructokinase, 3-Hydroxypropionic acid

## Abstract

**Background:**

*Escherichia coli* have both the Embden–Meyerhof–Parnas pathway (EMPP) and Entner–Doudoroff pathway (EDP) for glucose breakdown, while the EDP primarily remains inactive for glucose metabolism. However, EDP is a more favorable route than EMPP for the production of certain products.

**Results:**

EDP was activated by deleting the *pfkAB* genes in conjunction with subsequent adaptive laboratory evolution (ALE). The evolved strains acquired mutations in transcriptional regulatory genes for glycolytic process (*crp*, *galR*, and *gntR*) and in glycolysis-related genes (*gnd*, *ptsG*, and *talB*). The genotypic, transcriptomic and phenotypic analyses of those mutations deepen our understanding of their beneficial effects on cellulosic biomass bio-conversion. On top of these scientific understandings, we further engineered the strain to produce higher level of lycopene and 3-hydroxypropionic acid.

**Conclusions:**

These results indicate that the *E*. *coli* strain has innate capability to use EDP in lieu of EMPP for glucose metabolism, and this versatility can be harnessed to further engineer *E. coli* for specific biotechnological applications.

**Supplementary Information:**

The online version contains supplementary material available at 10.1186/s13068-022-02219-6.

## Background

Bacterial glucose metabolism is particularly diverse and involves the orchestrated actions of multiple pathways, including the Embden–Meyerhof–Parnas pathway (EMPP), Entner–Doudoroff pathway (EDP), pentose phosphate pathway (PPP), and oxidative pathway (via gluconic acid) [1, 2]. These pathways produce pyruvate, reducing power (NADH and/or NADPH), and energy (ATP), and play a central role in growth and bio-production [3, 4]. In contrast to *Escherichia coli,* which primarily uses the EMPP for glucose metabolism, *Pseudomonas,* and *Zymomonas* spp. employ the EDP, as they lack phosphofructokinase (Pfk), a pivotal EMPP enzyme. The PPP is a metabolic pathway that functions in parallel with either the EMPP or EDP and supplies the energy requirements for cell growth, NADPH regeneration, and replenishment of essential precursors for nucleotide and amino acid synthesis.

The EMPP comprises 10 enzymatic steps, yielding two pyruvate, two net ATP, and two NADH molecules per one glucose molecule, whereas the EDP utilizes only five enzymes to have two pyruvates (one of which is produced from glyceraldehyde-3-phosphate (G3P) via the lower glycolysis), one net ATP, one NADH, and one NADPH molecule per one glucose molecule. The EDP has been reported to have certain advantages compared with the EMPP [5]. For example, it is the thermodynamically more favorable pathway, owing to lower ATP production, in turn, boosting glycolysis [6]. Considering the number of enzymatic steps involved, the costs of protein synthesis in EDP is lower than that of EMPP [7]. It is also characterized by less allosteric regulation due to the lack of Pfk catalysis, one of the committed steps in the EMPP, which is found to be subjected to intensive regulation at the transcription, translation, and even post-translation and allosteric levels via distal metabolites, as well as energetic precursors [8]. The EDP is also associated with higher NADPH production to provide high reducing power for biosynthesis, resistance to oxidative stress, and the optimal synthesis of both the carbon and redox precursors for terpenoid biosynthesis. For example, *Z*. *mobilis* that relies extensively on the EDP shows notably rapid glucose uptake and ethanol production rates [9], and *Pseudomonas* species that utilizes an incomplete EMPP shows high production of highly toxic chemicals associated with their innate high solvent tolerance. Besides, the absence of a Pfk step facilitates the cyclization of PPP, which regenerates excess NADPH [10, 11].

Nevertheless, although there are several distinct benefits of employing EDP, the industrial strains of *E*. *coli* do not use their inherent EDP for glucose metabolism. Several studies have been conducted to engineer *E*. *coli* in an effort to activate the intrinsic EDP and to block the EMPP to promote more efficient biosynthesis. Previously, blockage of the EMPP via deletion of the phosphoglucose isomerase gene (*pgi*) resulted in re-routing the glycolytic flux through the PPP and EDP. The Δ*pgi* recombinant *E*. *coli* strains were characterized with enhanced production of polyhydroxybutyrate [12], isoprenoid [13], and hydrogen [14]. However, although the Δ*pgi* mutants remain viable, given that the glycolytic flux is re-routed through the PPP and EDP, excess NADPH production was found to perturb a significant portion of the metabolic network, resulting in an approximately 80% reduction in cell growth in glucose minimal media when compared with the wild-type levels [15]. Disrupting the EMPP by knocking out the phosphofructokinase I (PfkA) gene also resulted in re-routing the glycolytic flux through the PPP (~ 60% of the glycolytic flux) and the native EDP (~ 14% of glycolytic flux) [16]. The Δ*pfkA* recombinant *E*. *coli* strains showed enhanced production of 1,3-diaminopropane [17], hydrogen and ethanol [18], lycopene [19], and methyl 3-hydroxybutyrate [20]. However, these strains showed a more significant decrease in growth rate compared with the Δ*pgi* mutants owing to the partial cyclization of PPP, which may produce excess NADPH [21]. *E. coli* in which the EMPP was completely blocked by deleting *pfkAB* showed retarded growth on glucose minimal medium due to redox imbalance and reduced glucose uptake, suggesting the PPP and EDP were not sufficiently activated to alleviate the metabolic defect.

In this study, we isolated and characterized a *pfkAB*-deleted *E*. *coli* mutant strains that can thrive on glucose minimal medium with adaptive laboratory evolution (ALE). Using these strains, we investigated the genotype–phenotype relationship to determine and classify the requirements for the full activation of the EDP in *E*. *coli*. Finally, as a proof of concept and based on the preliminary information obtained, we further engineered the evolved mutant to confer it with the ability to produce a higher level of 3-hydroxypropionic acid (3-HP) that requires more NADPH regeneration.

## Results and discussion

### Adaptive evolution of the ∆*pfkAB* strain and analysis of mutations identified in the evolved strains

**Fig. 1:**
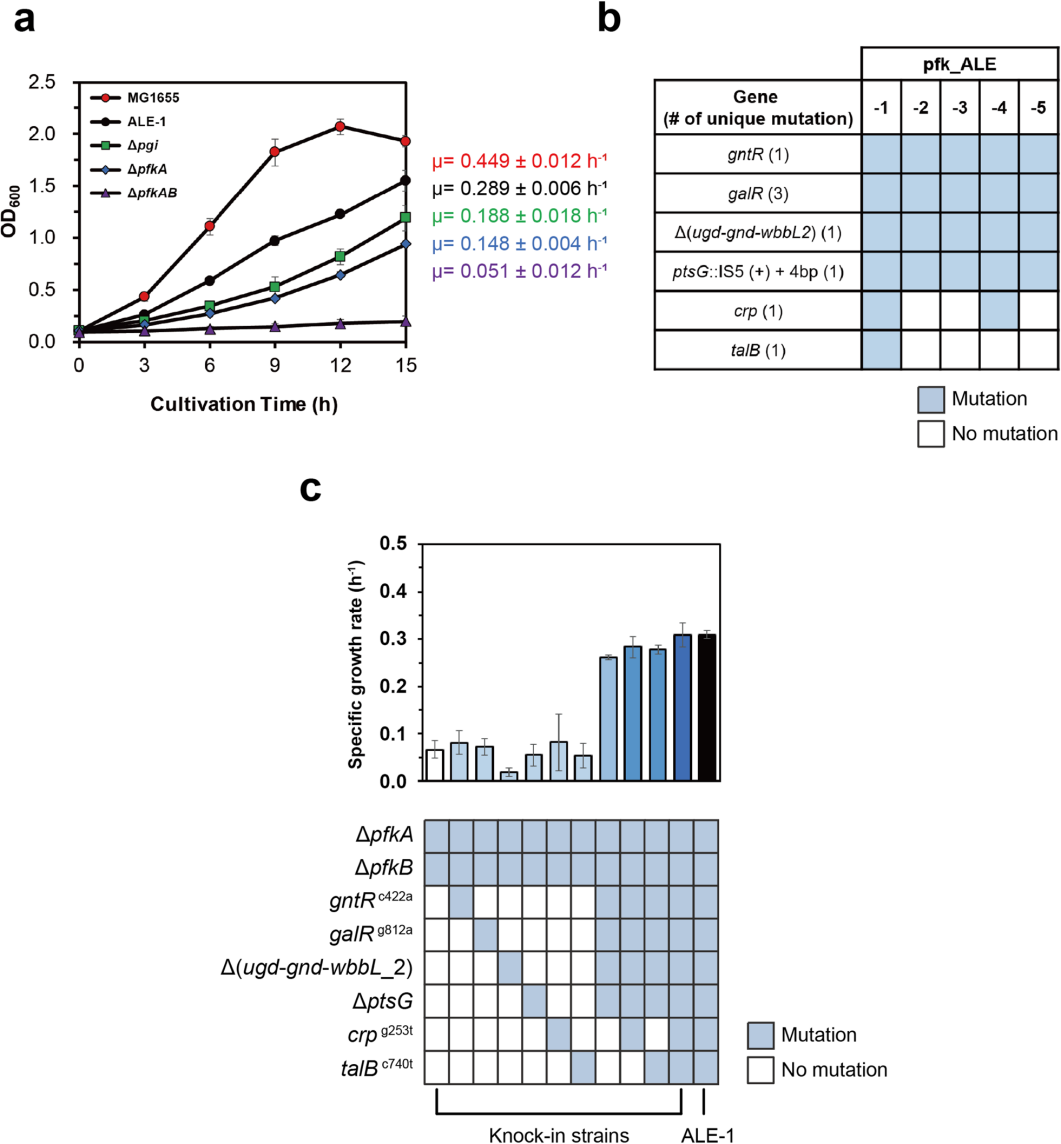
Comparison of cell growth on glucose among EMPP-disrupted strains and the effect of mutations on cell growth of the evolved strains and their background strains. **a** Growth rate profiles (OD_600_) were compared for three groups of *E. coli* strains: wild type with MG1655, complete disruption of EMPP with background strain (∆*pfkAB*), and evolved strain (ALE-1), and partial disruption of EMPP with the phosphofructokinase I step (∆*pfkA*) and phosphoglucose isomerase step (∆*pgi*). Error bars indicate standard deviations of measurements for three independent biological replicates. **b** Genetic characterization of five independent evolved ∆*pfkAB* isolate strains with genome resequencing analysis. The number of unique mutations per gene is noted within brackets. **c** The effect of mutations on cell growth were determined by introducing single or multiple mutations in the background strain ∆*pfkAB*. For the mutation in *ptsG*, the IS5 insertion disrupted the protein, and consequently, PtsG would lose its functionality. Thus, we knocked out the *ptsG* gene instead of knocking-in the IS element. Error bars indicate standard deviations of growth measurements for three independent biological replicates

Given that *E*. *coli* has two Pfk isozymes, Pfk-I and Pfk-II encoded by *pfkA* and *pfkB*, respectively [22], a recombinant *E*. *coli* strain with complete inactivation of the EMPP was constructed by deleting both of *pfkA* and *pfkB* genes. The ∆*pfkAB* strain showed slower specific growth rate on minimal medium containing glucose as a sole carbon source (Fig. 1a). ALE experiments were performed to recover the reduced cell growth by activating the EDP. After 50 subcultures of the ∆*pfkAB* strain in M9 minimal medium supplemented with 5 g/L glucose, the strain thereafter grew more rapidly on glucose. The five evolved ∆*pfkAB* strains showing recovered cell growth were isolated and denoted as pfk*_*ALE-1, -2, -3, -4, and -5 strains (Additional file 1: Figure S1a). The ALE-1 exhibited 92% of cell mass accumulation of the wild-type MG1655 strain after culturing for 20 h. This is the first *E*. *coli* strain with a completely inactivated EMPP that can grow more rapidly than mutants with partial blockage of the EMPP, such as the ∆*pfkA* and ∆*pgi* mutants (Fig. 1a). Besides, the ALE-1 strain showed improved terpenoid biosynthesis via the methylerythritol phosphate pathway, which has been observed in EDP-activated *E*. *coli* strains, because the synthetic pathway starts with 1-deoxy-d-xylulose 5-phosphate (DXP) synthase reaction with the two substrates G3P and pyruvate that are the final products of EDP. In addition, the synthetic pathway uses NADPH as sources of reducing regent produced by EDP. Interestingly, the production culture conditions used in the present study were able to rescue cell growth of the ALE-1 strain, with a 115% higher cell mass accumulation than the wild type at 36 h of culturing (Additional file 1: Figure S2).

We sequenced the whole genome of ALE-1 and compared the sequence with the parental strain, *E*. *coli* MG1655. Resequencing identified six mutations in ALE-1 (Table 1). To confirm enriched and major mutations during ALE, the mutations detected in the ALE-1 were analyzed in the rest of four evolved mutants by PCR amplification and DNA sequencing. Mutations were found in the *gntR*, *galR, ptsG*, and *ugd-gnd-wbbL-2* sequences of all five pfk_ALE strains, whereas mutations in the *talB* and *crp* genes were detected only in one or two strains (Fig. 1b). Notably, *galR* showed three unique mutations during adaptive evolution, but all resulted in truncated and non-functional protein (Additional file 1: Figure S1b, c). This suggests that the *galR* mutation could be critical for the growth of the evolved strain. In contrast, mutation in the *talB* gene was only observed in the ALE-1 strain and *crp* mutation was detected in the ALE-1 and ALE-4 strains, probably indicating that these mutations might not considerably affect the physiological background during ALE [23–25].

**Table 1:** List of mutations identified in the evolved strain pfk_ALE-1

Gene	Gene product and its size (number of aa)^*a*^	Type of mutation^*b*^	Change^*c*^ in protein and nucleotide
*gntR*	Gluconate repressor protein (331)	SNP	A141E (GCG → GAG)
*galR*	Galactose repressor protein (343)	SNP	G271D (GGC → GAC)
*ptsG*	Glucose-specific PTS enzyme IIBC (477)	INS	IS5 insertion (IS5( +) + 4 bp, coding 815–818/1434 nt)
*ugd*	UDP-glucose 6-dehydrogenase (388)	DEL	Truncated protein (∆176 bp, coding 992–1167/1167 nt)
*gnd*	6-Phosphogluconate dehydrogenase (468)		Loss of function (∆1407 bp, cording 1–1407/1407 nt)
*wbbL-2*	Interrupted rhamnosyltransferase (pseudogene)		Loss of function (∆349 bp, coding 1–349/349 nt)
*crp*	cAMP receptor protein (210)	SNP	A85S (GCA → TCA)
*talB*	Transaldolase B (317)	SNP	A247V (GCA → GTA)

^*a*^*aa*, amino acid, *UDP* uridine 5′-diphosphoglucose, *cAMP* cyclic adenosine monophosphate

^*b*^*SNP* single nucleotide polymorphism, *INS* insertion, *DEL* deletion

^*c*^ + , insertion; *bp* base pairs, *nt* nucleotide, ∆ deletion

To compare the effects of the above mutations on the growth of ALE-1 strain, single mutations or combinations of the multiple mutations were introduced into the background Δ*pfkAB* strain. The growth rates of the resulting strains were compared with that of the background and evolved strain. The single mutation in the *gntR*, *galR*, *crp* caused an increase in the specific growth rate of 1.2-fold, 1.1-fold, and 1.2-fold, respectively, relative to the background Δ*pfkAB* strain. The single mutation in *talB* and the *ptsG* deletion mutant of the background strain showed a slight decrease in the specific growth rate. The background strain carrying a deletion of the *ugd-gnd-wbbL-2* region showed an even slower cell growth. When each of the six mutations was introduced independently, the restored cell growth levels of the resulting mutants were considerably lower than that of the evolved strain. Eventually, when the mutant was harboring all six mutations, the growth rate was comparable to ALE-1 (Fig. 1c). Based on these observations, we deduced that the enhanced growth differentiating ALE-1 from the background mutant was attributed not only to the occurrence of a single mutation in *gntR*, *galR*, *ugd-gnd-wbbL-2*, *ptsG*, *crp,* and *talB*, but also to their synergistic interaction.

GntR is a DNA-binding transcriptional regulator that represses the expression of genes involved in gluconate metabolism in the absence of the effector molecule D-gluconate [26]. Gluconate is phosphorylated by gluconate kinase to produce 6PG, which is also produced from glucose. It is finally metabolized to G3P and pyruvate via the 6-phosphogluconate dehydratase (Edd) and 2-keto-3-deoxy-6-phosphogluconate aldolase (Eda) enzymes of EDP. Knockout of *gntR* has previously revealed that the expression of *edd* and *eda* genes can occur even in the absence of gluconate [27]. Therefore, we hypothesized that the loss-of-function mutation in the *gntR* gene de-represses the originally latent genes of the EDP, thereby providing a major route for glucose metabolism in ALE-1 with an inactive EMPP (Δ*pfkAB*) and an inactive oxidative PPP (Δ*gnd*) (Table 1) [28]. In fact, ALE-1 Δ*edd* strain could not grow on glucose, indicating that ALE-1 relies on the EDP on glucose as the sole carbon source (Additional file 1: Figure S3).

GalR is a transcriptional regulator that down-regulates the genes associated with galactose metabolism when this sugar is unavailable [29]. The regulon of GalR harbors the *galP* gene encoding galactose permease, which also has glucose uptake activity [30]. Wild type *E*. *coli* mainly takes up external glucose by a phosphoenolpyruvate-dependent phosphotransferase system (PTS), which consists of enzyme I (PtsI), HPr (PtsH), and glucose-specific PTS EIIA (Crr) and EIICB (PtsG) components [31]. Thus, we anticipated that the evolved mutant that lost the PTS activity due to PtsG inactivation by insertion of the IS5 and SgrS regulation of sugar transporter mRNAs (*ptsG* and *manXYZ*) due to the accumulation of glucose-6-phosphate, and consequently, the strain can co-metabolize glucose and xylose, which was the case in the *ptsG*-deleted *E*. *coli* strains (Additional file 1: Figure S7d–f) [32–34]. It has also been reported that the loss of PtsG function results in retarded cell growth due to the limited capacity of glucose import, and non-PTS glucose transporters such as GalP can restore the glucose uptake rate in *ptsG* null mutants [35, 36]. Based on this assumption, we hypothesized that the expressed GalP, which is depressed by the inactivation of *galR,* rescues the glucose uptake capacity of ALE-1.

Moreover, another possible reason for the expression of a non-PTS glucose permease GalP in EMPP-disrupted strains could be the low availability of phosphoenolpyruvate (PEP), which is essential for the PTS-dependent glucose uptake. Previous studies showed that the ∆*pfkA* mutant had reduced PEP concentration [15]. Besides, an increase in flux through the EDP can further lower the intracellular PEP pool [16]. It is supported by the stoichiometry of the EDP, where PEP generation per glucose is half of that of the PPP or EMPP [13]. Limited glucose importing capacity due to low PEP availability can be supplemented by the expression of PEP-independent glucose permease and glucokinase [35].

cAMP receptor protein (CRP) is a global transcription regulator that directly or indirectly regulates the expression of over 100 genes [37–39]. The mutation site of CRP^A85S^ was very close to the cAMP binding site, suggesting the possible effect on the binding affinity. However, most of cAMP-independent CRP mutants contained mutations away from the binding site such as I112, T127, and A144 residues [40]. Therefore, it is hard to establish that the CRP^A85S^ is a cAMP-independent mutant. Since no significant difference in the cell growth was observed by this mutation, the activity of this enzyme was not affected (Fig. 1c). However, the exact mechanism underlying the *crp* mutation-mediated growth rate enhancement in the evolved strain remains unclear. We discovered that *crp* mutation occurred in the later period of adaptation (during 30–50 repeats of culture) while *ptsG* mutation was detected in the earlier period of adaptation (during 0–30 repeats of culture). Although PTS inactivation by *ptsG* mutation is an efficient way to trigger carbon catabolite repression (CCR) release like *galP* expression, the modification of binding between CRP and cAMP might further contribute the release of CCR and provide a strong selective pressure during adaptation [41].

TalB is an enzyme involved in the non-oxidative PPP and provides a reversible reaction between the PPP and the upper EMPP [42]. The A247 residue is located in an α-helix and away from the active center of *E. coli* TalB. The B-factor value of the mutated residue was kept considerably lower, indicating that the mutation stably maintains its α-helix structure. In addition, the substrate binding affinity of the TalB^A247T^ in *E. coli* W3110 strain was not modified in a previous study [43]. Moreover, the expression level of the *talB* gene in ALE-1 was not significantly changed (1.3-fold) by this mutation compared to that in MG1655 (Additional file 1: Figure S5). Since the ALE-1 strain lacks the *gnd* gene, a change in the non-oxidative PPP flux might be directed by a genetic mutation in *talB* during adaptation to supply precursors essential for growth.

### Functional analysis of the genetic mutations with transcriptomic and phenotypic data

**Fig. 2:**
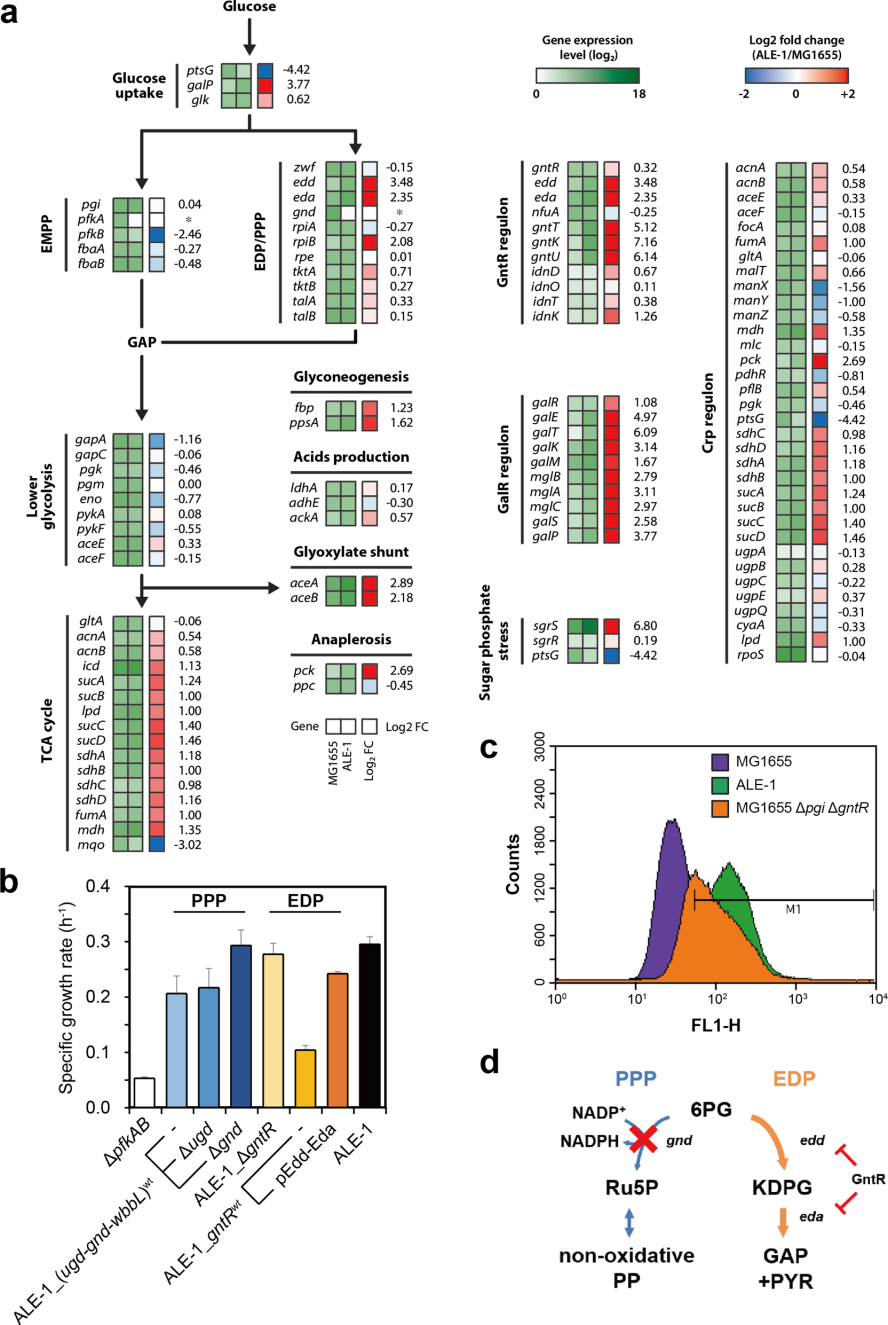
Functional analysis of major mutations found in the evolved strains. **a** RNA-seq transcriptome analysis illustrated the expression level of genes involved in glucose uptake system, glycolytic pathway, TCA cycle and transcriptional regulons including *gntR* and *galR*, *crp* regulons and sugar phosphate stress. Expression change (log_2_ fold change) of *pfkA* and *gnd* genes was unable to be calculated due to loss of expression and it was marked with an asterisk (*). Unexpected expression level of the *pfkB* gene of ALE-1 was affected by a remained coding sequence of *pfkB* (Additional file 1: Figure S8). **b** Functional validation experiments identified effective target genes among mutated genes from ALE-1: lacking the Gnd function, and loss of function for GntR and de-repression of its target genes, *edd* and *eda*. Error bars indicate standard deviations of growth measurements for three independent biological replicates. **c** Combined fluorescence histograms of MG1655, ALE-1, MG1655 Δ*pgi* Δ*gntR* strains carrying pHexR-GFP. **d** The schematic diagram illustrates altered glucose metabolism in ALE-1 through the EDP, via enzymes encoded by *edd* and *eda*. Transcriptional repression by GntR is indicated with red marks

Functional analysis of the mutations detected in the ALE-1 strain was conducted to elucidate the molecular mechanisms underlying the effects of these mutations. We performed transcriptome analysis with RNA-seq to investigate the transcriptomic effects of mutations involved in the glycolytic pathway and global transcriptional regulation. Transcriptomic analysis revealed the expression levels of 4497 genes on the chromosome of MG1655 and ALE-1 strains. Among those genes, we first focused on the glycolytic pathways including the EMPP, EDP and PPP. Complete deletion of the *pfkA* gene was confirmed with no *pfkA* expression. In ALE-1, the expression of the *edd* and *eda* genes, which are involved in the EDP, were 11.2- and 5.1-fold higher than in MG1655, respectively. This indicates that the latent EDP was activated during adaptation due to loss of function for GntR. Additionally, the expression level of the GntR regulon in ALE-1 was higher compared to MG1655. There was no expression of *gnd*, involved in the oxidative PPP, due to its complete deletion in ALE-1 (Fig. 2a). Taking these observations into consideration, the ALE-1 strain utilizes glucose via the EDP rather than the EMPP and oxidative PPP since ALE-1 lacks functional PfkA, PfkB, GntR, and Gnd. The RNA-seq analysis also showed lower expression level of *ptsG* in ALE-1 compared to MG1655, caused by degradation of *ptsG* mRNA. Instead, the expression level of the non-PTS glucose transporter, *galP*, in ALE-1 was 13.7-fold higher than MG1655. This phenomenon was also confirmed by quantitative RT-PCR, demonstrating 7.6-fold increased expression level (Additional file 1: Figure S5). Notably, we found that the expression levels of the *galR* regulon in ALE-1 was higher than that in MG1655 due to the inactivation of GalR (Fig. 2a). These indicate that the limited glucose uptake capacity was rescued by the GalP via the inactivation of GalR. However, only a small change was observed in the *gntR* and *galR* expression level between the MG1655 and ALE-1 strains, which suggests that the transcriptional levels of *gntR* and *galR* were not affected by the genetic mutation. Instead, mutations in *gntR* and *galR* might result in loss of protein function since the expression levels of their regulon were down-regulated. During evolution, ALE-1 activated the latent pathways, including the glyoxylate shunt and phosphoenolpyruvate carboxykinase (Pck) reaction. Since the glyoxylate shunt does not regenerate NADPH unlike the isocitrate dehydrogenase (Icd) reaction, the evolved strain might enhance the glyoxylate shunt flux rather than the Icd reaction to reduce the NADPH regeneration capacity. Overall, transcriptome analysis suggested that the glycolytic flux was re-routed through the activation of latent pathways and rescued the glucose uptake capacity in ALE-1. On the other hand, even though glucose can be utilized via mannose PTS [41], the expression levels of *manX*, *manY*, and *manZ* in ALE-1 were 2.9-, 2.0-, and 1.5-fold reduced compared to those in MG1655, respectively (Fig. 2a).

To further confirm our hypotheses, we initially deleted the *gntR*, *galR*, and *ptsG* genes in the ALE-1 strain. We found that the deletion of either the *gntR*, *galR* or *ptsG* gene did not affect the growth of ALE-1 cells, whereas restoration of these mutations resulted in notable adverse effects on the growth of ALE-1 cells (Fig. 2b). These results imply that GntR and GalR mutants may be unable to repress the expression of their target genes, including *edd*, *eda*, and *galP*. As expected, the same phenotype was observed in the ALE-1 strain that had a transposon inserted in *ptsG* or had a deleted *ptsG* mutation, confirming that the *ptsG* gene with the IS insertion lost its activity. To verify the target genes of the two TFs, we expressed the plasmid-encoded endogenous *edd*-*eda* or *galP* gene in the *gntR*- and *galR*-restored ALE-1, respectively, resulting in increased growth from 36 and 55% up to 82% and 86% of ALE-1, respectively. These observations thus indicate that the plasmid-based expression of *edd*-*eda* and *galP* genes can functionally recover cell growth defects caused by the restored active GntR and GalR in ALE-1 (Fig. 2b and Additional file 1: Figure S4a). To verify the effect of the large deletion of the *ugd-gnd-wbbL-2* region carrying the two functional genes *ugd* and *gnd* on ALE-1 cell growth, we re-introduced the deleted region into the chromosome of ALE-1. The restored strain showed reduced cell growth, and then the deletion of the *gnd* gene, but not the *ugd* gene, resulted in a substantial recovery of the growth defect caused by the restoration of the *ugd-gnd-wbbL-2* region (Fig. 2b and Additional file 1: Figure 4a).

According to the transcriptome analysis, we hypothesized that the ALE-1 uses EDP instead of the EMPP or oxidative PPP as the major glycolytic pathway. Since 2-keto-3-deoxy-6-phosphogluconate (KDPG) is an intermediate of EDP, intracellular KDPG level should be higher in ALE-1 compared to its wild-type strain. It was previously reported that the transcription of *hexR*, that originated from *P*. *putida* KT2440, was repressed in the presence of KDPG produced from glucose or gluconate [44]. To analyze the EDP flux, a KDPG-responsible biosensor using a transcriptional regulator (HexR) and GFP was constructed to sense the intracellular level of KDPG. Using an appropriate gate, M1, 85.8% of the population of ALE-1 and 9.5% of the wild-type MG1655 were detected (Fig. 2c). Previously, the glycolytic pathway of Δ*pgi* Δ*gntR* double-knockout *E. coli* strain was redirected from EMPP to EDP [28]. In fact, 64.2% of the population of the Δ*pgi* Δ*gntR* double-knockout cells were detected with M1 gate, which was higher than wild-type strain. This KDPG-responsible biosensor successfully supported our hypothesis that the EDP of ALE-1 was more active than that of the wild-type.

In conclusion, the growth of the EMPP-deficient mutant was facilitated by a number of factors that includes, 1) the re-direction of the glucose flux from the oxidative PPP to EDP by the deletion of *gnd*, 2) the gain of the EDP activity by the lack of *gntR*, and 3) the recovery of glucose uptake by the inactivation of GalR (Fig. 2d and Additional file 1: Figure S4b).

### Prioritization between the EDP and PPP for optimal cell growth

**Fig. 3:**
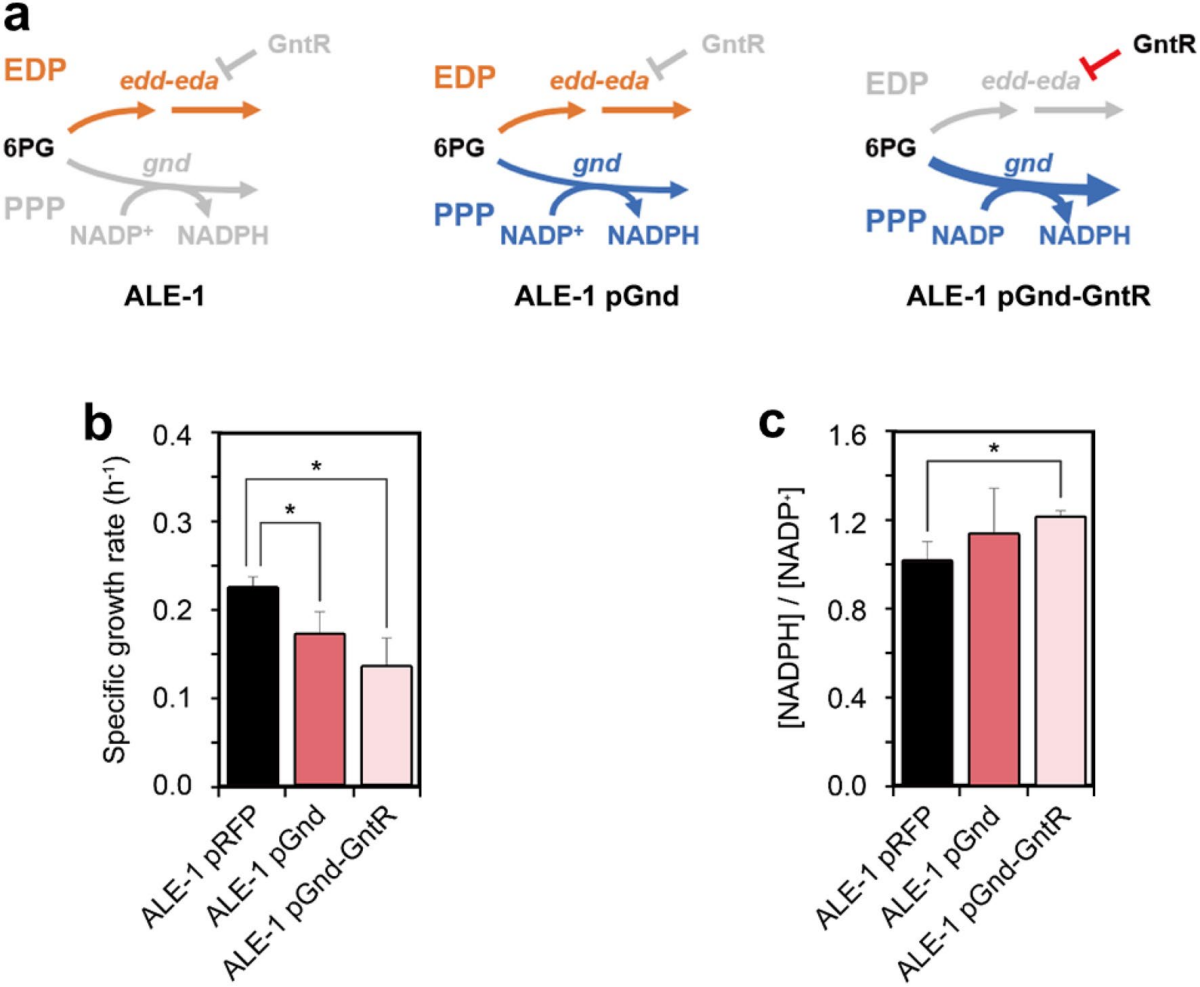
The effect of the redistribution of metabolic flux between PPP and EDP on cell growth and the NADPH/NADP ratio. **a** Schematic diagrams for possible glycolytic flux redistribution in ALE-1 pRFP (active EDP), ALE-1 pGnd (active both PPP and EDP), and ALE-1 pGnd-GntR (more active PPP and weaker EDP). The effect of gradually increasing glycolytic flux redistribution from the EDP to the PPP in the evolved ALE-1 strain on (**d**) cell growth and **e** [NADPH]/[NADP^+^] ratio. Error bars indicate standard deviations of growth measurements for three independant biological replicates. An asterisk (*) indicates a significant difference compared to the ALE-1 pRFP (*p* < 0.05)

Notably, the evolved strain had the *gnd* mutation resulting in the inactivation of oxidative PPP, an alternative glycolytic pathway. The ALE-1 strain thus employs only the EDP for glycolysis, indicating that an active PPP might harm the growth of bacterial cells using the EDP as the main glycolytic pathway. The availability of the ALE-1 strain with plasmid-based Gnd expression enabled us to assess the effect of glucose catabolism via both oxidative PPP and EDP on its cell growth (Fig. 3a, b). As expected, expression of Gnd conferring oxidative PPP activity together with the EDP had an adverse effect on the growth of ALE-1 cells, leading to a 23% decrease in the growth rate of the evolved strain. We further investigated the effect of the further increased glycolytic flux from the EDP to oxidative PPP on the growth of ALE-1 cells by expressing both *gnd* and *gntR* on a plasmid, where Gnd reactivates oxidative PPP and GntR represses the EDP (Fig. 3a, c). As a result, high glycolytic flux to oxidative PPP resulted in more severe growth defects, resulting in a 39% reduction in cell growth, but exhibiting a 120% higher NADPH/NADP^+^ ratio (Fig. 3d, e). Using HexR-based KDPG-responsible biosensor, we further examined the activity of EDP in ALE-1 with plasmid-based expression of the *gnd* and/or *gntR* genes to confirm that expression of those genes led to redirect glycolytic flux. As a result, the concentration of intracellular KDPG was decreased when only *gnd* was expressed and further decreased when both the *gnd* and *gntR* were expressed (Additional file 1: Figure S6). This observation led us to speculate about why the oxidative PPP function was lost instead of being conserved to handle the glycolytic flux together with the EDP. It has been suggested that one of the reasons why the *E*. *coli* Δ*pgi* mutant, which is primarily dependent on the PPP for glucose catabolism, shows reduced glucose uptake rate is due to a redox imbalance and/or an excess of NADPH regenerated from the PPP [45]. When glucose is metabolized via the EDP, one less NADPH is regenerated than via the PPP in this mutant [46]. However, the PPP of the *pfkAB* knockout mutant can regenerate excess NADPH even more intensively due to its cyclized architecture compared with the *pgi*-null mutant [10]. Taking these observations into consideration, the effect of re-routing the glycolytic flux to the EDP on the reduction of the regeneration of NADPH would be more significant in the latter mutant. Despite partial relief afforded by the EDP, the NADPH formed via the cyclized PPP might exceed the demand for cell growth.

Moreover, the EDP has been known to be thermodynamically favorable, even when compared with the native glycolytic route EMPP. This thermodynamically favorable property enables the EDP to sustain a glycolytic flux comparable to that of the EMPP [6, 7]. Indeed, *Z*. *mobilis*, which extensively employs the EDP for glycolysis, takes up glucose at a notably rapid rate [9]. This observation indicates that the capacity and turnover rate of the EDP would be substantial [47, 48]. Considering the foregoing observations, establishing the EDP as the sole glycolytic route, rather than sharing the flux with the PPP, would benefit the growth of the Pfk-deficient mutant in terms of both reducing NADPH production and expanding glycolytic capacity during selection. Consistent with this hypothesis, the soil bacterium *P*. *putida* KT2440, which lacks a functional EMPP, is known to be almost extensively dependent on the EDP for glycolysis, with less than 10% of the glucose flux entering the PPP [2].

### Dynamic regulation of the EDP and PPP for biotechnological applications

**Fig. 4:**
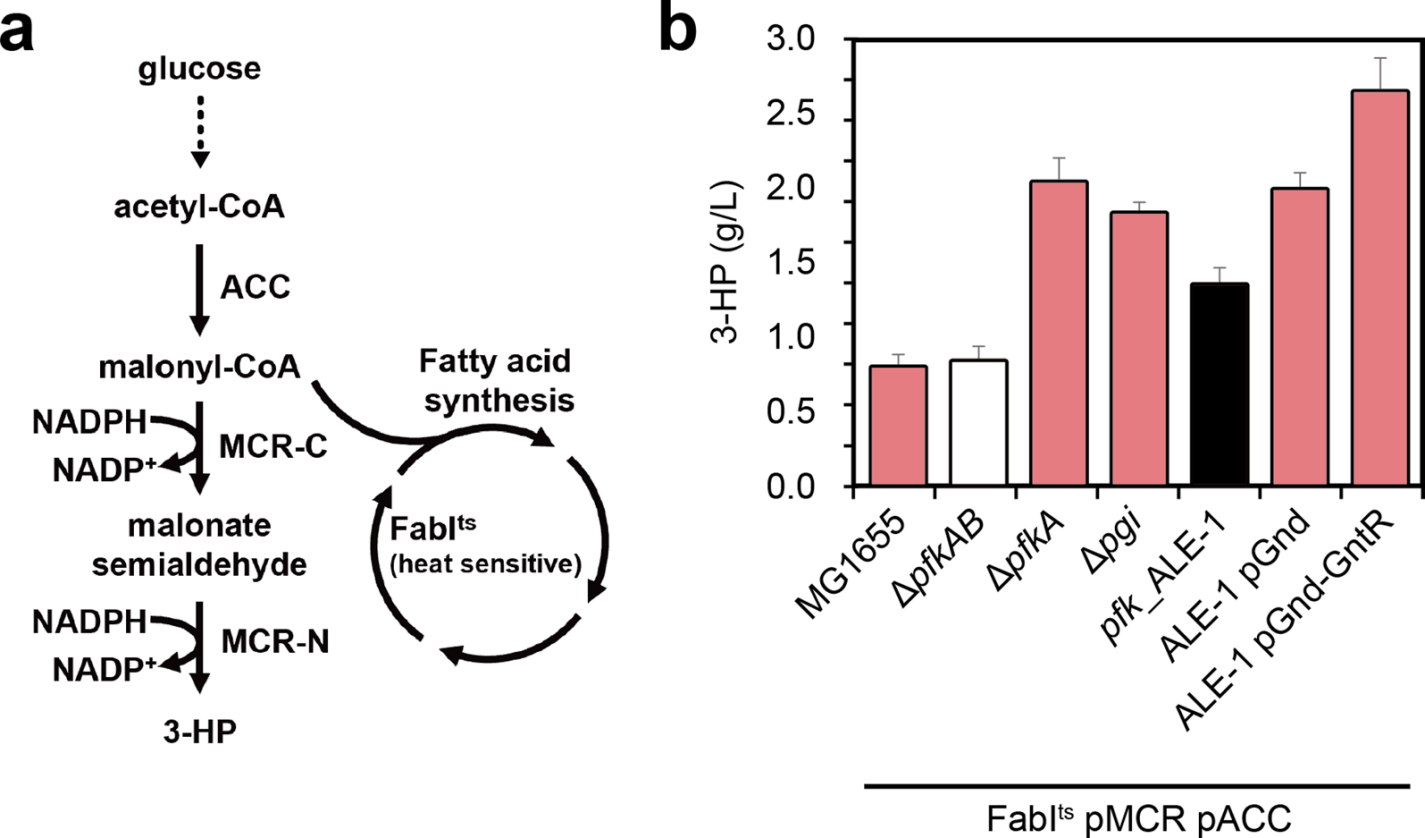
Rebalancing carbon flux through the PPP and EDP for enhanced 3-HP production. **a** A diagram for genetic strategy for the efficient production of 3-HP. A heterologous 3-HP synthetic pathway was introduced by expressing two dissected subparts, N-terminus and C-terminus ones, from the mutant malonyl-CoA reductase gene (*mcr*) derived from *C*. *aurantiacus*. ACC was overexpressed for malonyl-CoA accumulation while fatty acid synthesis was reduced by expressing a temperature-sensitive FabI at 37 °C. **b** 3-HP production titer at 48 h post-induction. Error bars indicate standard deviations of 3-HP titer for three independent biological replicates, respectively

To facilitate optimal growth, cells must stringently balance their intracellular redox. However, the microbial production of value-added chemicals often requires an abundant supply of reducing powers in the form of NADPH. Accordingly, there exists a fundamental difference in the optimal architecture of the core metabolic pathways to sustain normal growth and those required for metabolite overproduction. For the successful application of the evolved mutant described herein for bio-production, further manipulation of the core metabolic pathways would be necessary. For proof of concept, we introduced changes into the evolved strain ALE-1 machinery for it to attain an abundant supply of NADPH necessary for the production of 3-HP, an intermediate for many compounds. The bifunctionality of 3-HP enables it to be further transformed into a variety of high-value compounds [49–51]. From 1 mol of glucose, it is theoretically possible to generate 2 mol of 3-HP with a consumption of 4 mol of NADPH (Fig. 4a). However, its redox equivalent demand is far beyond the endogenous catabolic supply based on glucose catabolism via the EDP or the linear PPP, which regenerates 1 or 2 mol of NADPH per mol of glucose [13]. According to the stoichiometry of the EDP and PPP, restoration of cyclized PPP activity and the reduction of EDP activity might yield larger amounts of 3-HP [10, 13]. To address this issue, we demonstrated the conditional flux control towards the EDP and PPP at the 6PG node for the enhanced production of 3-HP.

Before dealing with the redox issue, we attempted to eliminate a potential limiting factor, namely, the low availability of malonyl-CoA for 3-HP production. Efficient microbial production of chemicals such as flavonoids and fatty acids using malonyl-CoA as a precursor are often constrained by the low intracellular concentrations of this compound [52]. In the present study, we overexpressed acetyl-CoA carboxylase (ACC) to enhance the supply of malonyl-CoA, and in conjunction performed site-directed mutagenesis designed to increase the temperature sensitivity of endogenous enoyl-[acyl-carrier-protein] reductase (FabI), thereby reducing the malonyl-CoA consumption via fatty acid synthesis [53, 54] (Fig. 4a). At 48 h post-induction, the evolved strain ALE-1 produced 1.34 g/L of 3-HP, whereas the background strain produced 0.83 g/L. Although the evolved mutant produced a 1.6-fold higher 3-HP titer than the background strain under the same conditions, the fold-increase was subtle, considering there was a substantial difference in biomass accumulation between the two strains (Fig. 1a and Fig. 4b).

To achieve conditional flux control towards the EDP and PPP, the *E*. *coli gnd* and *gntR* genes were expressed under the control of an IPTG-inducible promoter, and the resulting pGnd and pGnd-GntR plasmids were transformed into ALE-1 harboring the 3-HP-producing machinery. Induction of flux redistribution to both the EDP and PPP in the 3-HP-producing ALE-1 mutant harboring pGnd led to a considerable enhancement in the 3-HP titer (1.99 g/L) at 48 h post-induction. Furthermore, when the EDP was repressed by additional expression of its repressor GntR, 3-HP production in the evolved mutant increased further to 2.65 g/L, which is 3.2- and 3.3-fold higher than the titer obtained from the background mutant and wild-type *E*. *coli* strains, respectively, harboring the same 3-HP synthesis machinery (Fig. 4b and Additional file 1: Figure S2). These findings thus indicate that facilitating glucose flux via the PPP and reducing the flux through the EDP are critical for generating an abundant NADPH supply, resulting in higher 3-HP production.

## Conclusions

In this study, we generated and characterized an engineered *E*. *coli* using EDP rather than EMPP as the major glycolytic pathway, whose pathway is not functional in the wild-type strain. Based on genetic and biochemical studies, we demonstrated that optimal cell growth of *E*. *coli* strain through the EDP requires three main metabolic changes: (1) de-repression of the EDP by inactivation of GntR, (2) inactivation of the oxidative PPP by deletion of Gnd, and (3) an increase in glucose uptake by the inactivation of GalR. Reverse engineering of the evolved strain was applied to enhance the production of 3-HP by increasing glycolytic flux from the EDP to PPP for the supply of excess NADPH during the production stage rather than the growth stage. The engineered strain and its derivatives have certain advantages with respect to specific bio-production purposes, such as 3-HP and lycopene production, which demand NADPH (Fig. 4b and Additional file 1: Figure S7a, b).

Two EMPP-disrupted *E*. *coli* mutants, in which the pathway was blocked at the Pfk or Pgi step, resulted in distinct genotypic changes after ALE with glucose as the sole carbon source [18, 23, 55]. The evolved *E*. *coli* strain lacking *pfkA* showed a mutation in the promoter region of *pfkB* coding for a minor isozyme of Pfk. The expression of Pfk increased with time and this enabled the strain to restore glucose metabolism through the EMPP and could support cell growth. This observation indicates that, among three glycolytic pathways (EMPP, EDP, and PPP), the EMPP may be the most conducive for maximal cell growth in *E*. *coli* strains. The loss of Pgi led to a significant increase in relative flux through the PPP. None of the evolved Δ*pgi* mutants could influence the absolute EDP flux. Instead, independent studies have revealed that mutations leading to enhanced activity of transhydrogenase have repeatedly been detected in adapted Δ*pgi* mutants, suggesting that these mutations may reduce NADPH imbalance [23, 55–57]. In the present study, we found that the activation of the latent EDP by de-repressing downregulation was one of the key fitness-enhancing factors during the adaptation of the Pfk-null mutant. These findings imply that there are fundamental differences in the metabolic apparatus of the Pfk- and Pgi-null mutants, although both are widely used to redirect the glycolytic flux to the EMPP. One of them is that PPP can be cyclized and produce more NADPH in the Pfk-null mutant.

Although slightly slower growth was found in ALE-1 compared with the wild-type strain that employs the EMPP as the main glycolytic pathway, the evolved strain generated in this study grew well on glucose minimal medium compared with *pgi-* or *pfkA-*deleted strains that are characterized by a partially inactive EMPP. An additional factor that may contribute to the suboptimal growth of the evolved strain is that an imbalance or excess of NADPH produced from EDP can reduce cell growth when glucose is used as the sole carbon source. Notably, *E*. *coli* expressing G6PDH is strictly NADP^+^-specific compared with that in other bacteria that primarily employ the EDP as the glycolytic pathway [58–60]. Previous studies have reported that the growth of the *pgi* mutant could not be restored fully to that of the wild type rate by overexpressing UdhA for the re-oxidation of NADPH, indicating that the transhydrogenase system is not sufficient to overcome an imbalance in NADPH [45]. When NADPH-consuming pathways (lycopene and 3-HP production) were engineered in ALE-1, we observed the recovery of cell growth; the lycopene- and 3-HP-producing ALE-1 cell showed higher cell growth than the wild type. These findings suggest that the evolved strain could be a suitable host cell for NADPH-dependent bio-production, as slightly reduced cell growth minimally affects production.

The selection of a suitable host cell for the production of a biotechnology-based product is crucial to ensure economic viability. The bacterium, *E*. *coli*, is one of the best-characterized microorganisms from the genetic and metabolic perspectives and has a variety of tractable traits for biotechnological production. However, tolerance to adverse environmental stress, or unfavorable redox potentials may impede the use of *E*. *coli* as a platform host organism. The selection of host cells for production should be based on several factors, including the availability of genetic tools for expressing target products, efficient utilization of alternative carbon sources, high redox potential, and a tolerance to the metabolic products. Furthermore, unlike *Pseudomonas* and *Zymomonas*, ALE-1 can co-metabolize glucose and xylose, which was the case in the *ptsG*-deleted *E*. *coli* strains (Additional file 1: Figure S7d–f). ALE-1 strain even grows under anoxic conditions using nitrate as a final electron acceptor (Additional file 1: Figure S7c).

In conclusion, we have demonstrated that the normally latent EDP in *E*. *coli* can be almost fully activated by enhancing glucose uptake through an inactivation of GalR, inactivating the EDP regulatory system, and blocking the glycolytic flux via the PPP. The resulting strain and its derivatives have advantageous traits that are found in microorganisms using the EDP and PPP as the major glycolytic route, and it is amenable to host strong redox reactions. The engineered strains could expand the utility of *E*. *coli* as a platform for biotechnological production.

## Materials and methods

### Reagents, strains and media

Unless otherwise indicated, the reagents used in this study were purchased from Sigma–Aldrich (St. Louis, MO, USA).

The bacterial strains and plasmids used in this study are listed in Additional file 1: Table S1. The wild-type *E*. *coli* MG1655 strain was used as a parent strain for the generation of mutant strains. The *E*. *coli* DH10B strain was used for bacterial transformation and plasmid amplification during the construction of the expression plasmids.

Unless otherwise stated, as growth media, we used M9 minimal medium (BioShop Canada Inc., Burlington, Canada) supplemented with 2 mM MgSO_4_, 0.1 mM CaCl_2_, and glucose with appropriate antibiotics, if required, and Luria–Bertani (LB) medium. The LB medium was prepared using 5 g/L yeast extract, 10 g/L peptone and 10 g/L NaCl. Antibiotics were used at the following concentrations: ampicillin (100 mg/L), kanamycin (50 mg/L), and chloramphenicol (30 mg/L).

### Cell culture

For growth measurements, a single colony from an agar plate was inoculated in 3 mL of LB medium at 37 °C and shaken at 200 rpm. Overnight-grown cells were 1:10 diluted into 5 mL of M9 medium supplemented with 4 g/L glucose. After growing for 12 h, cells were inoculated into 25 mL of M9 medium in 250-mL shaking flask at 37 °C and shaken at 200 rpm to achieve an initial optical density at 600 nm (OD_600_) of approximately 0.1. For functional analysis, a single colony was cultivated in 3 mL of LB medium with appropriate antibiotics at 37 °C and shaken at 200 rpm overnight. A seeded culture was 1:10 diluted into 5 mL of M9 medium supplemented with 4 g/L glucose and appropriate antibiotics. After 12 h in culture, cells were diluted into 25 mL of M9 medium in a 250 mL shaking flask to achieve an initial OD_600_ of 0.1. The expression of *galP* and *edd*-*eda* was induced by the addition of 6.25 µM of isopropyl-β-D-thiogalactopyranoside (IPTG) at the beginning of seeding, subculturing, and culturing. The expression of pRFP, pGnd, and pGnd-GntR plasmids was induced by the addition of 50 µM of IPTG after 3 h in culture. OD_600_ was measured using a Libra S22 spectrophotometer.

For 3-HP production, a single colony from an agar plate was used to inoculate 3 mL of LB medium at 30 °C and shaken at 200 rpm. Overnight-grown cells were 1:20 inoculated into M9 medium supplemented with 20 g/L glucose, 1.0 g/L yeast extract, 2 mM MgSO_4_, 0.1 mM CaCl_2_, and trace elements. The composition of the trace element solution used was as follows: 2.4 g of FeCl_3_·6H_2_O, 0.3 g of CoCl_2_·6H_2_O, 0.15 g of CuCl_2_·2H_2_O, 0.3 g of ZnCl_2_, 0.3 g of Na_2_MO_4_·2H_2_O, 0.075 g of H_3_BO_3_, and 0.495 g of MnCl_2_·4H_2_O per liter [54]. After growing at 30 °C and shaken at 200 rpm for 9 h, 50 µM of IPTG and 100 nM of tetracycline were added to induce the expression of mcrC*-*mcrN*, and *accDABC*, after which the temperature of the culture was increased to 37 °C.

### Adaptive laboratory evolution

To isolate rapidly growing mutants, even those lacking a functional EMPP, a mutant in which both the *pfkA* and *pfkB* genes had been deleted was adapted by sequential transfer. Initially, an overnight culture of ∆*pfkAB* mutant cells in LB was transferred to M9 minimal medium supplemented with 2 mM MgSO_4_, 0.1 mM CaCl_2_ and 5 g/L glucose and grown at 37 °C and shaken at 200 rpm. At an OD_600_ of approximately 1.0, the cells were passaged to a 250-mL shaking flask containing 25 mL of the same fresh M9 medium. This procedure was repeated 50 times, after which, the adapted cells were plated onto an LB agar plate. Five evolved mutants showing the most rapid biomass accumulation were selected based on OD_600_ values monitored using a 96-well microplate reader and named pfk_ALE-1, -2, -3, -4, and -5 (henceforth referred to as ALE-1, -2, -3, -4, and -5).

### Genome sequencing

Genomic DNA was isolated from the evolved ALE-1 strain using a DNA isolation kit (GeneAll Biotechnology Co., Korea), and subsequently sequenced via next-generation sequencing using an Illumina HiSeq 2000 sequencer (Macrogen Inc., Korea). The genomic sequence of *E*. *coli* MG1655 (NC_000913.3) was used as a reference. Read sequence data of wild-type and ALE-1 strains were deposited in the Sequence Read Archive (SRA) of the NCBI under BioProject accession number PRJNA818725, and BioSample accession number SAMN26878847 for wild-type strain, SAMN26878848 for ALE-1 strain. The occurrence of mutations was confirmed by DNA sequencing of the PCR amplicon of target regions in the background strain and evolved mutant. To analyze the mutation frequency, the presence of mutations that were either the same or equivalent to the mutations discovered in ALE-1 was analyzed in the other four ALE mutants by PCR amplification and DNA sequencing.

### Genetic manipulation

Gene knockout was conducted by homologous recombination via a λ RED recombination system, which was mediated by a kanamycin resistance (Km^R^) cassette flanked by Flp recognition target (FRT) knock-in and flippase-based curing [61, 62]. Knockout constructs were designed with 50-bp overhangs homologous to upstream and downstream sequences of the deletion site. Cells were screened for antibiotic sensitivity, and for those lacking antibiotic resistance, removal of the Km^R^ gene was confirmed by colony PCR and sequencing.

Point mutations were generated via oligonucleotide-mediated genome editing combined with a *tetA*-based dual selection system. The *tetA* cassette was inserted into the target site and replaced with oligos containing target mutations and short 50-bp overhangs homologous to sequences upstream and downstream of the insertion site. The recombinants were isolated by negative selection using 50 μM NiCl_2_. Cells were screened for antibiotic sensitivity, and for those lacking antibiotic resistance, the correct allelic replacement was confirmed by colony PCR and sequencing.

### Plasmid construction

All plasmids constructed in this study were derived from BioBrick plasmids [63]. The primers used for plasmid cloning are listed in Additional file 1: Table S2. The primers were synthesized by Macrogen Inc. (Seoul, Korea). Plasmid construction was conducted using Pfu-X DNA Polymerase for the PCR reactions, and all restriction enzymes used were purchased from Fermentas (Vilnius, Lithuania). For plasmid isolation, we used a LaboPass plasmid miniprep kit (Cosmo Genentech, Korea). DNA purification (Cat. no.103-102) and gel extraction (Cat. no.102-102) kits were purchased from GeneAll Biotechnology (Seoul, Korea).

For the construction of pEdd-Eda, the gene encoding the red fluorescent protein (RFP) in the pBbA6c-*rfp* vector was excised using restriction enzymes, *Eco*RI and *Bam*HI. The *edd-eda* gene cluster was amplified from *E*. *coli* MG1655 genomic DNA using a forward primer containing an *Eco*RI site and a reverse primer containing a *Bam*HI site. Following excision with *Eco*RI and *Bam*HI, the purified *edd-eda* fragment was ligated with the gel-eluted pBbA6c backbone.

For cloning the pGalP, the RFP-encoding gene in the pBbS6k-*rfp* vector was excised using restriction enzymes, *Bgl*II and *Xho*I. The *E*. *coli galP* gene was amplified from *E*. *coli* MG1655 genomic DNA using a forward primer containing a *Bgl*II site and a reverse primer containing an *Xho*I site. Following excision with restriction enzymes, *Bgl*II and *Xho*I, the purified *galP* fragment was ligated with the gel-eluted pBbS6k backbone.

To construct pGnd, the RFP-encoding gene in the pRFP vector was excised using *Eco*RI and *Xho*I. The *E*. *coli gnd* gene was amplified from the *E*. *coli* MG1655 genomic DNA with a forward primer containing an *Eco*RI site and a reverse primer containing an *Xho*I site. Following excision with *Eco*RI and *Xho*I, the purified *gnd* fragment was ligated with the gel-eluted pRFP backbone.

For the construction of pGnd-GntR*,* the *E*. *coli gntR* gene was amplified from *E*. *coli* MG1655 genomic DNA with a forward primer containing a *Bgl*II site and a reverse primer containing the *Xho*I site. Following excision with restriction enzymes, *Bgl*II and *Xho*I, the purified *gntR* fragment was ligated with the purified pGnd.

For the construction of the pMCR plasmid, a mcrC^*^-*mcrN* fragment including genes encoding the dissected C-terminal domain carrying three mutations (N391V/K557W/S565N) and the N-terminal domain of malonyl-CoA reductase from *Chloroflexus aurantiacus* was extracted after cutting the pCDF-mcrC*-*mcrN* plasmid at *Eco*RI and *Xho*I sites. The pBbE2k-*rfp* plasmid was digested with restriction enzymes, *Eco*RI and *Xho*I to excise the gene encoding RFP, after which the DNA fragment and the gel-eluted pBbE2k backbone were ligated.

For the construction of a pACC plasmid, a fragment harboring the *accDABC* gene cluster encoding the ACC complex was obtained by elution after cutting pBbA2k-*accDABC* at *Bgl*II and *Xho*I sites. The pBbA6c-*rfp* plasmid was digested with restriction enzymes, *Bgl*II and *Xho*I to excise the gene encoding RFP. After gel-elution, the pBbA6c backbone was ligated with the eluted *accDABC* fragment.

### Transcriptome analysis (RNA-seq)

Cell culture samples were harvested at the mid-log growth phase (OD_600_ ~ 0.5 for MG1655 and ALE-1 strain). The RNA was stabilized using by RNAprotect^®^ Bacteria Reagent (Qiagen #76506) and total RNA was isolated using RNeasy^®^ Plus Mini Kit (Qiagen #74134). Ribosomal RNA (rRNA) was removed from isolated RNA samples through riboPooL Probes (Pan-prokaryote) siTOOLS BIOTECH and Dynabeads^™^ MyOne^™^ Streptavidin C1(Invitrogen #65001). For purification of RNA samples, RNA clean & concentrator (zymoresearch R1013) was used. RNA library was constructed from rRNA-depleted RNA using the KAPA stranded RNA-seq kit (Roche KK8400). The sequencing libraries were sequenced by Nextseq 550, NextSeq 500/550 High Output Kit v2.5 (024906).

Using bowtie software, the sequence reads were mapped onto the wild-type reference genome (NC_000913.2) with the maximum insert size option of 1500 bp (for Additional file 1: Figure S8, 500-bp option was used) [64]. Fragments per kilobase of exon per million fragments (FPKM) and log_2_ fold change value were calculated by cufflinks [65]. All the datasets were visualized using Metascope and NimbleGen’s Signalmap software. The transcriptomic data of wild-type and ALE-1 strains were deposited in NCBI’s Gene Expression Omnibus (GEO) under GEO Series accession number GSE199153.

On the other hand, the unexpected expression level of the *pfkB* gene in ALE-1 was affected by a remaining coding sequence of *pfkB* (Additional file 1: Figure S8). Considering that the active site of PfkB was deleted, we anticipated that the function for PfkB in the ALE-1 strain was lost.

### Flow cytometric analysis

For cultivation, a single colony was inoculated in 3 mL of LB medium at 37 °C and shaken at 200 rpm. Overnight-grown cells were 1:50 diluted into 5 mL of M9 medium supplemented with 4 g/L glucose and 5 g/L yeast extract. After growing for 9 h, cells were collected and transferred to PBS. The KDPG-responsible biosensor was induced by the addition of 50 µM of IPTG at the beginning of seeding and culturing. The green fluorescent protein (GFP) fluorescence intensity of individual cells was measured by BD FACSCalibur Flow Cytometer (BD Bioscience, USA) with wavelength of 488 nm excitation. Nonbacterial particles were excluded by applying forward-scattered characteristics and side-scattered characteristics. Gate M1 was set to differentiate the population of cells based on fluorescence intensity. BD CellQuest Pro software was used to analyze the data and to create images.

### Analytical methods

Culture media were treated using previously reported methods with minor modifications [66]. After centrifuging for 30 min at 13,200 × *g*, the supernatant was heat-treated at 80 °C for 1 h. Thereafter, the supernatant obtained by centrifuging again for 30 min at 13,200 × *g* was used for analysis.

The concentrations of glucose and 3-HP were measured by HPLC using a previously described method with slight modification [67]. After a 20-fold dilution, 20 µL of samples were injected into a 300 mm × 7.8 mm Aminex HPX-87H (Bio-Rad, USA) column at 35 °C using 5.41 mM H_2_SO_4_ as the mobile phase.

## Supplementary Information

**Additional file 1: Figure. S1** Cell growth and description of *galR* mutations detected in pfk_ALE-2, -3, and -5 strains. (a) Growth rate profiles (OD_600_) were compared for the four evolved strains isolated after the 50th subculture during ALE. Growth rate profiles of Δ*pfkAB* and ALE-1 strains are presented as controls. (b, c) DNA sequence of wild-type and mutant *galR* coding region in ALE-2, -3 and -5 strains were compared. The sequences include the start codon (lower case), emerging site of early stop codon (grey box), duplicated target sequence (underlined), duplication (red), and insertion (blue). Adenine in the start codon of the *galR* gene is designated as +1. (b) A nonsense mutation was caused by a frame shift due to a 7-bp duplication in the *galR* gene of the ALE-2, -3 strains, which resulted in truncation of the C-terminal 33 amino acids of the total 344 amino acids. (c) Insertion of 768 bp and duplication of 8 bp in the *galR* gene of ALE-5 caused insertion of 259 amino acids between the 3rd and 4th amino acids of the wild-type GalR protein. These GalR mutants are expected to be equivalent to the GalR mutant of ALE-1, which has low binding affinity to target sequence in the absence of an appropriate inducer, galactose. **Figure. S2** Cell growth and 3-HP, glucose, and acetate concentration during 3-HP production. All strains have a *fabI* temperature-sensitive mutant (*fabI*^ts^) and two plasmids pMCR and pACC. Profiles of 3-HP concentration (red circles), cell mass (yellow triangles), consumed glucose concentration (green squares) and acetate (blue diamonds) were compared for *E. coli* strains: (a) MG1655 (b) MG1655 ∆*pfkA* (c) MG1655 ∆*pgi* (d) Δ*pfkAB* (e) ALE-1 (f) ALE-1 with pGnd and (g) ALE-1 with pGnd-GntR. Cell mass was calculated from the OD_600_ values. 1 OD_600_ unit corresponds to 0.33 g CDW/L [1]. Error bars indicate standard deviations of three independent biological replicates. **Figure. S3** The comparison of cell growth between pfk_ALE-1 and pfk_ALE-1 Δ*edd* strains. Growth rate profiles (OD_600_) of ALE-1 Δ*edd* strain was compared to that of ALE-1 strain on glucose as a sole carbon source. Cells were grown in 250 mL with 25 mL of M9 minimal medium containing 0.4% glucose. Error bars indicate standard deviations of three independent biological replicates. **Figure .S4** Functional analysis of mutations involved in glucose uptake system in the evolved strains. (a) Target genes affected by the mutated TF (GalR) or *ptsG* deletion were identified and their role for improved cell growth in ALE-1 were determined. Cells were grown in M9 minimal medium with 0.4% glucose in shaking flasks. IPTG (6.25 µM) was added at the beginning of seeding, subculturing, and culturing to induce each plasmid. Error bars indicate standard deviations of three independent biological replicates. (b) The schematic diagram illustrates the altered route of glucose uptake in ALE-1 via a non-PTS glucose transporter GalP, the expression of which is negatively regulated by GalR. **Figure. S5** Comparison of mRNA expression level of *galP* and *talB* in ALE-1 with MG1655. The mRNA expression level of *galP* and *talB* in MG1655 (gray bar) and ALE-1 (black bar) was analyzed by quantitative RT-PCR. Error bars indicate standard deviations of three independent biological replicates. **Figure. S6** Flow cytometric analysis of pHexR-GFP with expression of *gnd* and/or *gntR* genes at 9 hours cultivation. Fluorescence histograms of pHexR-GFP ALE-1, ALE-1 pGnd, and ALE-1 pGnd-GntR strain were compared. Gate M1 was used to differentiate the population of wild-type and EMPP-deficient strains based on GFP intensity. **Figure. S7** Diverse phenotypic characteristics of pfk_ALE-1. (a, b) Lycopene production was examined in ALE-1 and control strains all harboring the pAC-LYCO04 plasmid. (a) Lycopene titer of *E. coli* strains after 24 h of cultivation were compared. (b) Color of lycopene-producing strains after 24 h of cultivation. Two milliliters of lycopene-producing cells were harvested after 24 h of cultivation and re-suspended in 200 µL of distilled water. After transfer to 96-well plates, images of cells were taken using a 96-well microplate reader. (c) When nitrate was used as the final electron acceptor under anoxic conditions, OD_600_ of ALE-1 and MG1655 were measured. For OD_600_ of MG1655 with nitrate addition, there was no significant difference caused by nitrate addition (data not shown). (d–f) Residual sugar concentration and cell growth of ALE-1 and MG1655 strains when grown in M9 minimal media supplemented with 3 g/L of glucose and 2 g/L of xylose. Residual glucose and xylose concentration of (d) MG1655 and (e) ALE-1 strain. (f) The cell growth of both strains is presented. **Figure. S8** Unexpected expression level of the *pfkB* gene of ALE-1. In the process of deletion of the *pfkB* gene, nucleotides of 157bp from the 5′ end and 177bp from the 3′ end were detected. Using bowtie software, the sequence reads were mapped onto the wild-type reference genome (NC_000913.2) with the maximum insert size option of 500bp. Remained coding sequence of the *pfkB* gene affected the expression level of the *pfkB* gene of ALE-1. **Table S1.** Strains and plasmid list. **Table S2.** Primer List.

## Data Availability

All data generated or analyzed during this study are included in this published article and its Additional file 1 information files. Read sequence data of wild-type and ALE-1 strains were deposited in the Sequence Read Archive (SRA) of the NCBI under BioProject accession number PRJNA818725, and BioSample accession number SAMN26878847 for wild-type strain, SAMN26878848 for ALE-1 strain. The transcriptomic data of wild-type and ALE-1 strains were deposited in NCBI’s Gene Expression Omnibus (GEO) under GEO Series accession number GSE199153.

## Abbreviations

3-HP: 3-Hydroxypropionic acid
6PGDH: 6-Phosphogluconate dehydrogenase
ACC: Acetyl-CoA carboxylase
ALE: Adaptive laboratory evolution
ATP: Adenosine triphosphate
cAMP: Cyclic adenosine monophosphate
Crp: CAMP receptor protein
Eda: 2-Keto-3-deoxy-6-phosphogluconate aldolase
Edd: 6-Phosphogluconate dehydratase
EDP: Entner–Doudoroff pathway
EMPP: Embden–Meyerhof–Parnas pathway
FabI: Enoyl-[acyl-carrier-protein] reductase
FRT: Flippase recognition target
G3P: Glyceraldehyde-3-phosphate
GalP: Galactose permease
GalR: Galactose repressor protein
GFP: Green fluorescent protein
Gnd: 6-Phosphogluconate dehydrogenase
GntR: Gluconate repressor protein
HPLC: High-performance liquid chromatography
IPTG: Isopropyl-β-D-thiogalactopyranoside
LB: Luria–Bertani medium
MCR: Malonyl-CoA reductase
MEP: Methylerythritol phosphate
NAD^+^/NADH: Nicotinamide adenine dinucleotide (oxidized/reduced form)
NAD^+^/NADPH: Nicotinamide adenine dinucleotide phosphate (oxidized/reduced form)
OD_600_: Optical density (measured at a wavelength of 600 nm)
PEP: Phosphoenolpyruvate
Pfk: Phosphofructokinase
Pgi: Phosphoglucose isomerase
PPP: Pentose phosphate pathway
PTS: Phosphotransferase system
PtsG: Glucose-specific phosphotransferase system enzyme IIBC
SgrS: Sugar transport-related sRNA
TalB: Transaldolase B
TF(s): Transcriptional factor(s)
UDP: Uridine diphosphate
Ugd: UDP-glucose 6-dehydrogenase
WbbL_2: Interrupted rhamnosyltransferase
