# Supplementary material for: Characterization of an Entner–Doudoroff pathway-activated Escherichia coli
Source: Biotechnol Biofuels Bioprod. 2022 Nov 9;15:120. doi: 10.1186/s13068-022-02219-6 (PMC9648032; doi:10.1186/s13068-022-02219-6)
Supplement: Supplementary file 1 — Additional file 1: Figure. S1 Cell growth and description of galR mutations detected in pfk_ALE-2, -3, and -5 strains. (a) Growth rate profiles (OD600) were compared for the four evolved strains isolated after the 50th subculture during ALE. Growth rate profiles of ΔpfkAB and ALE-1 strains are presented as controls. (b, c) DNA sequence of wild-type and mutant galR coding region in ALE-2, -3 and -5 strains were compared. The sequences include the start codon (lower case), emerging site of early stop codon (grey box), duplicated target sequence (underlined), duplication (red), and insertion (blue). Adenine in the start codon of the galR gene is designated as +1. (b) A nonsense mutation was caused by a frame shift due to a 7-bp duplication in the galR gene of the ALE-2, -3 strains, which resulted in truncation of the C-terminal 33 amino acids of the total 344 amino acids. (c) Insertion of 768 bp and duplication of 8 bp in the galR gene of ALE-5 caused insertion of 259 amino acids between the 3rd and 4th amino acids of the wild-type GalR protein. These GalR mutants are expected to be equivalent to the GalR mutant of ALE-1, which has low binding affinity to target sequence in the absence of an appropriate inducer, galactose. Figure. S2 Cell growth and 3-HP, glucose, and acetate concentration during 3-HP production. All strains have a fabI temperature-sensitive mutant (fabIts) and two plasmids pMCR and pACC. Profiles of 3-HP concentration (red circles), cell mass (yellow triangles), consumed glucose concentration (green squares) and acetate (blue diamonds) were compared for E. coli strains: (a) MG1655 (b) MG1655 ∆pfkA (c) MG1655 ∆pgi (d) ΔpfkAB (e) ALE-1 (f) ALE-1 with pGnd and (g) ALE-1 with pGnd-GntR. Cell mass was calculated from the OD600 values. 1 OD600 unit corresponds to 0.33 g CDW/L [1]. Error bars indicate standard deviations of three independent biological replicates. Figure. S3 The comparison of cell growth between pfk_ALE-1 and pfk_ALE-1 Δedd strains. Growth r [file 13068_2022_2219_MOESM1_ESM.docx]

**Additional file 1 for**

**Characterization of an Entner-Doudoroff pathway-activated *Escherichia coli***

Ye Eun Kim^1,†^, Kyung Hyun Cho^1,†^, Ina Bang^1,†^, Chang Hee Kim^2^, Young Shin Ryu^1^, Yuchan Kim^1^, Eun Mi Choi^1^, Linh Khanh Nong^1^, Donghyuk Kim^1,2,*^, and Sung Kuk Lee^1,2*^

^1^School of Energy and Chemical Engineering, Ulsan National Institute of Science and Technology (UNIST), Ulsan 44919, Republic of Korea

^2^Department of Biomedical Engineering, UNIST, Ulsan 44919, Republic of Korea

^†^These authors contributed equally to this work.

*Corresponding author

Address: School of Energy and Chemical Engineering, Ulsan National Institute of Science and Technology (UNIST), Ulsan 44919, Republic of Korea.

Tel.: +82 52 217 2514; Fax: +82 52 217 2509

*E-mail address: sklee@unist.ac.kr (S.K. Lee)

**This file includes:**

Additional file 1: Figure S1 to Additional file 1 Figure S8

Additional file 1 Table S1 to Additional file 1 Table S2

Additional file 1 Material and Methods

**Additional file 1: Figures**

**a**


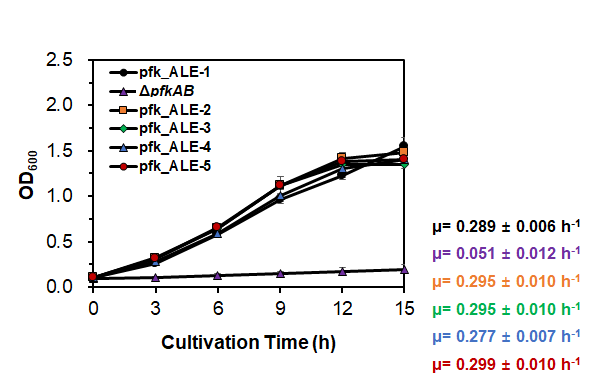


**c**

**b**


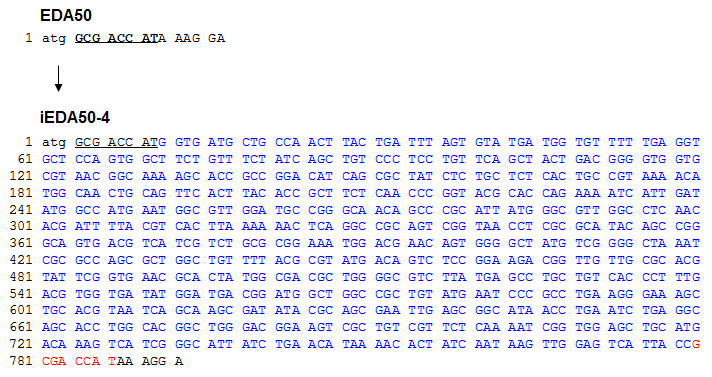

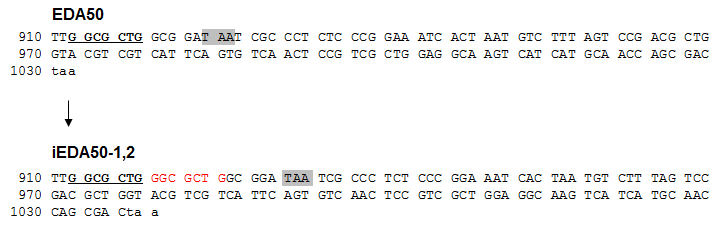


**pfk_ALE-1**

**pfk_ALE-2,3**

**pfk_ALE-1**

**pfk_ALE-5**

**Additional file 1 Figure S1. Cell growth and description of *galR* mutations detected in pfk_ALE-2, -3, and -5 strains.** (a) Growth rate profiles (OD_600_) were compared for the four evolved strains isolated after the 50^th^ subculture during ALE. Growth rate profiles of Δ*pfkAB* and ALE-1 strains are presented as controls. (b, c) DNA sequence of wild-type and mutant *galR* coding region in ALE-2, -3 and -5 strains were compared. The sequences include the start codon (lower case), emerging site of early stop codon (grey box), duplicated target sequence (underlined), duplication (red), and insertion (blue). Adenine in the start codon of the *galR* gene is designated as +1. (b) A nonsense mutation was caused by a frame shift due to a 7-bp duplication in the *galR* gene of the ALE-2, -3 strains, which resulted in truncation of the C-terminal 33 amino acids of the total 344 amino acids. (c) Insertion of 768 bp and duplication of 8 bp in the *galR* gene of ALE-5 caused insertion of 259 amino acids between the 3^rd^ and 4^th^ amino acids of the wild-type GalR protein. These GalR mutants are expected to be equivalent to the GalR mutant of ALE-1, which has low binding affinity to target sequence in the absence of an appropriate inducer, galactose.


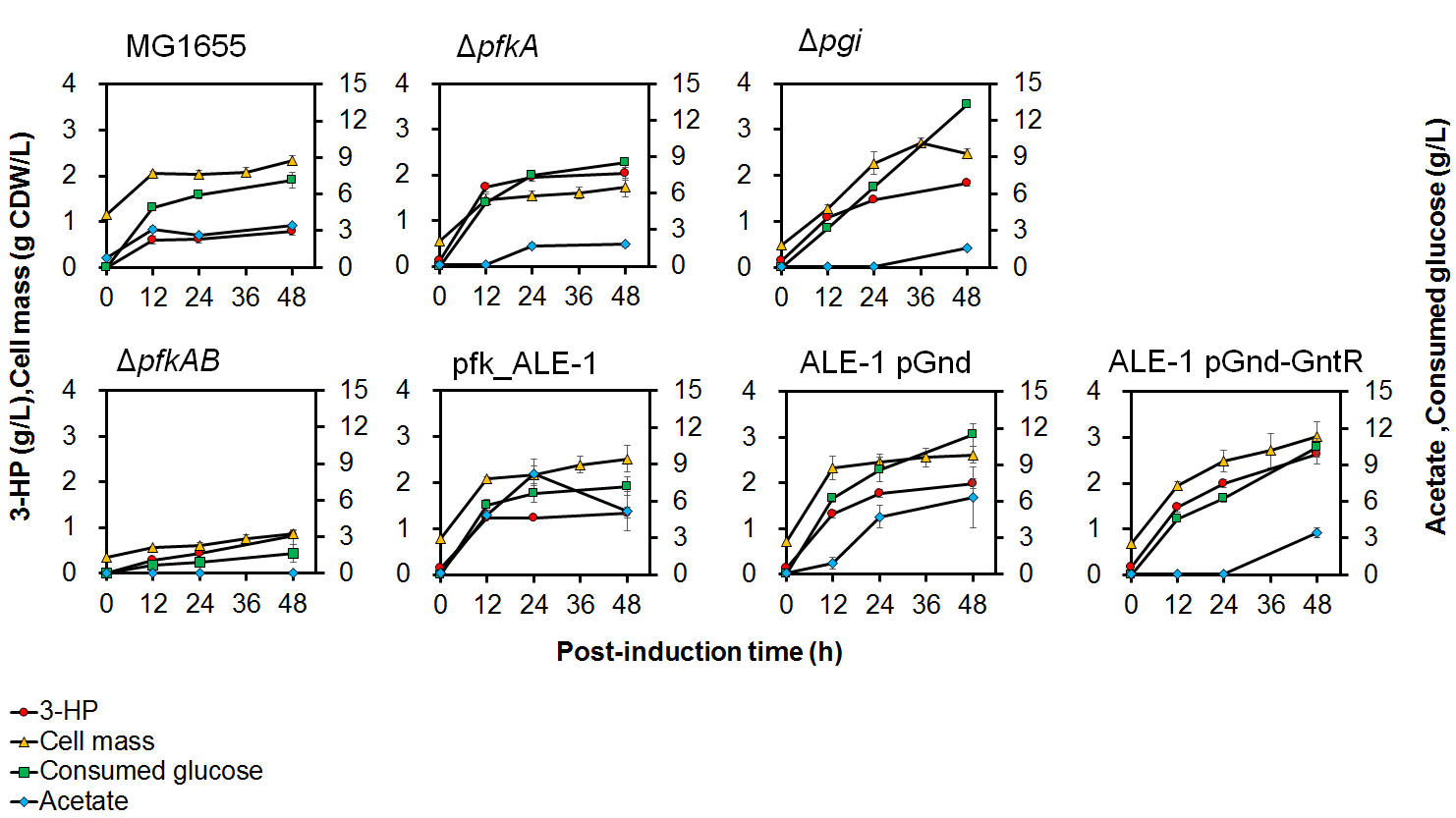


**a**

**b**

**c**

**d**

**e**

**f**

**g**


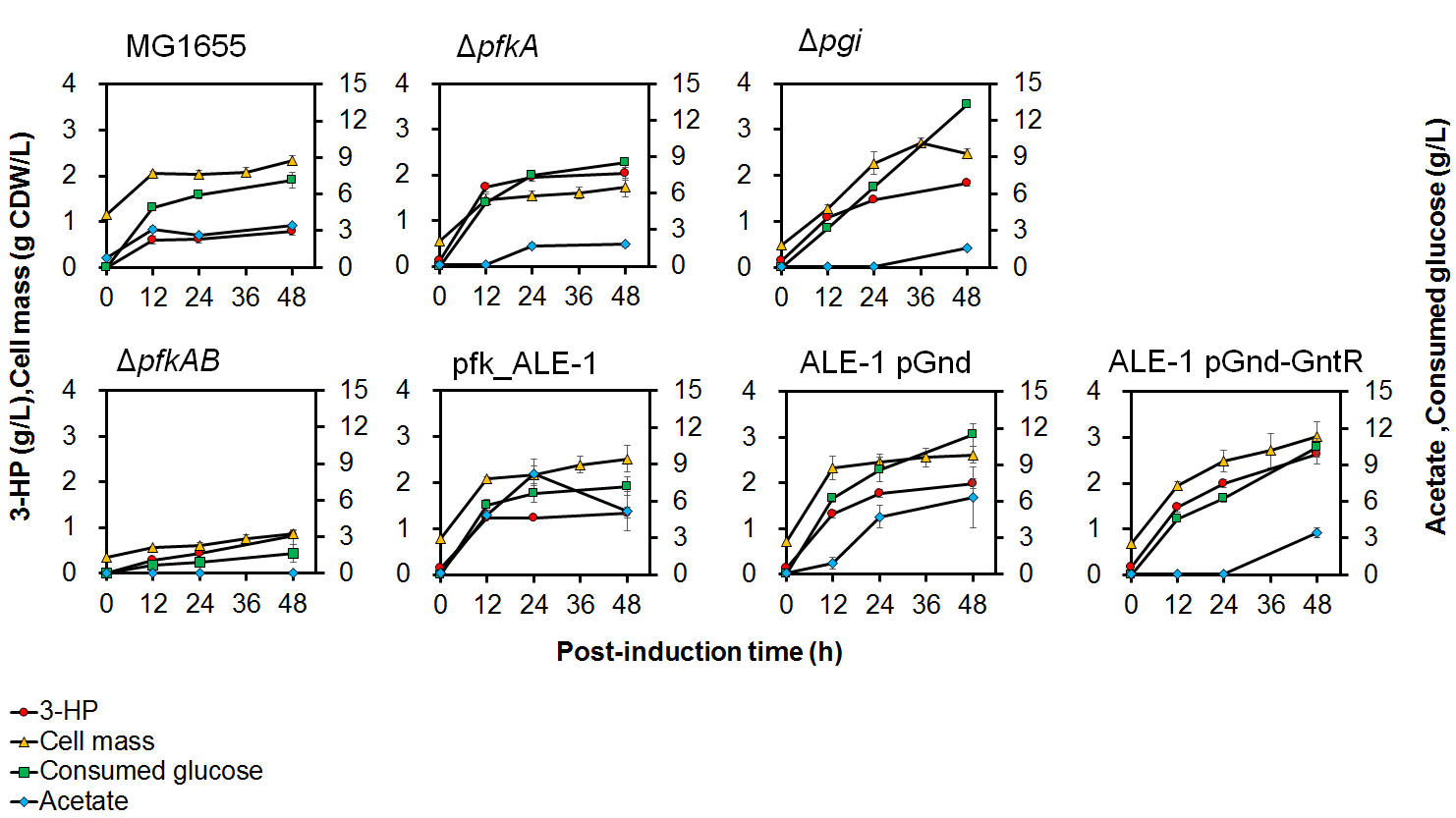


**Additional file 1 Figure S2. Cell growth and 3-HP, glucose, and acetate concentration during 3-HP production.** All strains have a *fab*I temperature-sensitive mutant (*fabI*^ts^) and two plasmids pMCR and pACC. Profiles of 3-HP concentration (red circles), cell mass (yellow triangles), consumed glucose concentration (green squares) and acetate (blue diamonds) were compared for *E. coli* strains: (a) MG1655 (b) MG1655 ∆*pfkA* (c) MG1655 ∆*pgi* (d) Δ*pfkAB* (e) ALE-1 (f) ALE-1 with pGnd and (g) ALE-1 with pGnd-GntR. Cell mass was calculated from the OD_600_ values. 1 OD_600_ unit corresponds to 0.33 g CDW/L [1]. Error bars indicate standard deviations of three independent biological replicates.


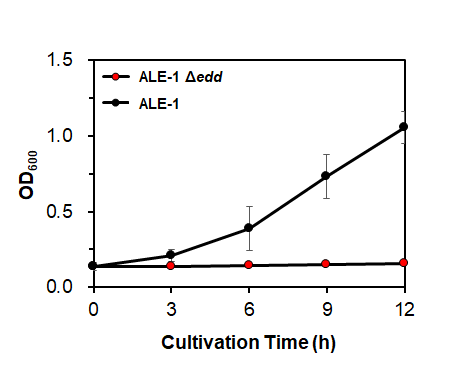


**Additional file 1 Figure S3. The comparison of cell growth between pfk_ALE-1 and pfk_ALE-1 Δ*edd* strains.** Growth rate profiles (OD_600_) of ALE-1 Δ*edd* strain was compared to that of ALE-1 strain on glucose as a sole carbon source. Cells were grown in 250 mL with 25 mL of M9 minimal medium containing 0.4% glucose. Error bars indicate standard deviations of three independent biological replicates.

**
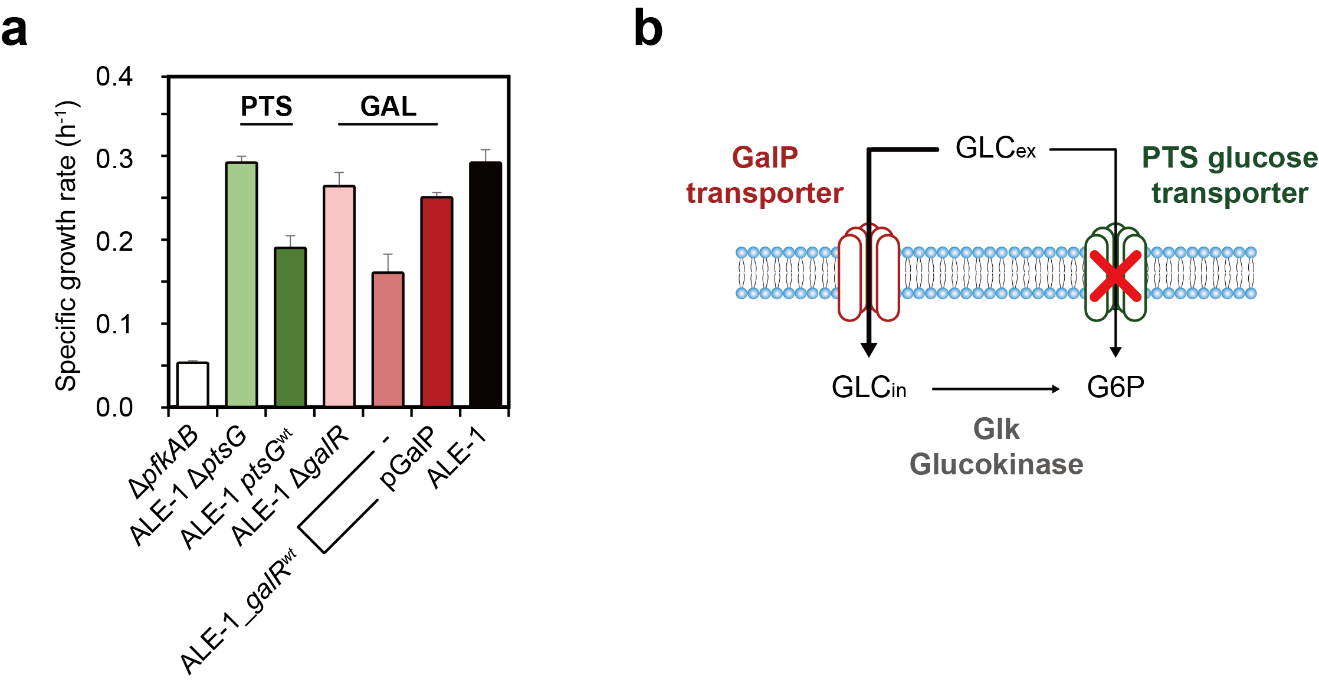
**

**Additional file 1 Figure S4. Functional analysis of mutations involved in glucose uptake system in the evolved strains.** (a) Target genes affected by the mutated TF (GalR) or *ptsG* deletion were identified and their role for improved cell growth in ALE-1 were determined. Cells were grown in M9 minimal medium with 0.4% glucose in shaking flasks. IPTG (6.25 µM) was added at the beginning of seeding, subculturing, and culturing to induce each plasmid. Error bars indicate standard deviations of three independent biological replicates. (b) The schematic diagram illustrates the altered route of glucose uptake in ALE-1 via a non-PTS glucose transporter GalP, the expression of which is negatively regulated by GalR.

**
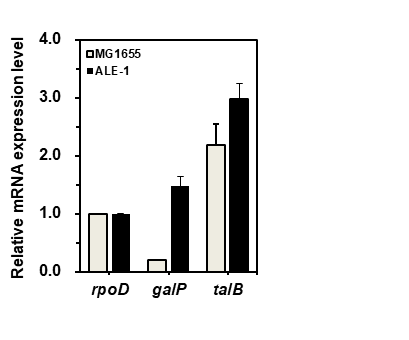
**

**Additional file 1 Figure S5. Comparison of mRNA expression level of *galP* and *talB* in ALE-1 with MG1655.** The mRNA expression level of *galP* and *talB* in MG1655 (gray bar) and ALE-1 (black bar) was analyzed by quantitative RT-PCR. Error bars indicate standard deviations of three independent biological replicates.


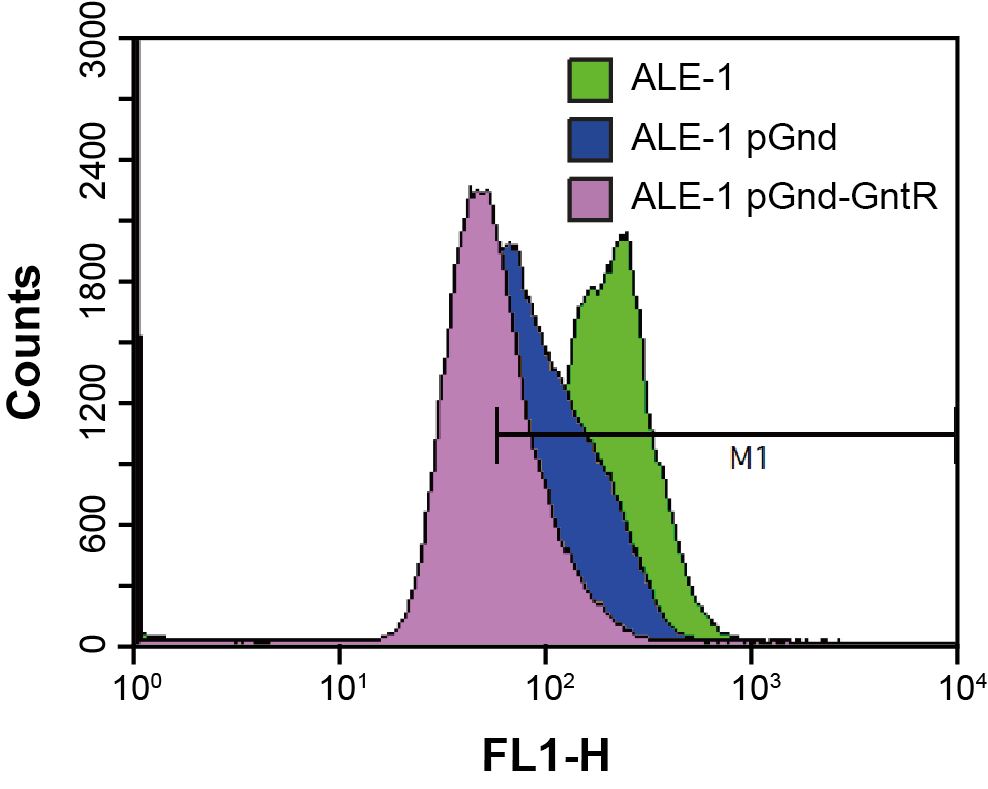


**Additional file 1 Figure S6. Flow cytometric analysis of pHexR-GFP with expression of *gnd* and/or *gntR* genes at 9 hours cultivation.** Fluorescence histograms of pHexR-GFP ALE-1, ALE-1 pGnd, and ALE-1 pGnd-GntR strain were compared. Gate M1 was used to differenciate the population of wild-type and EMPP-deficient strains based on GFP intensity.


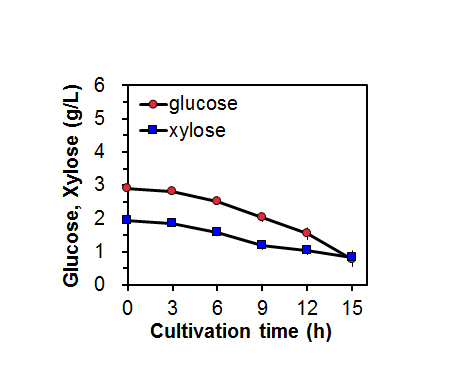

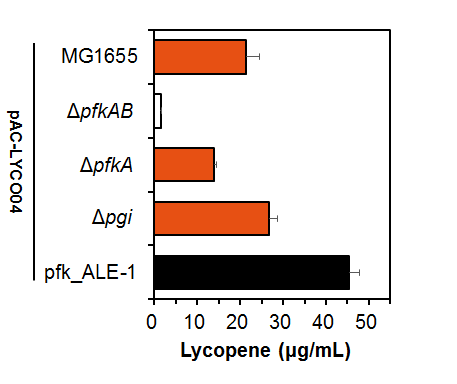


**a**


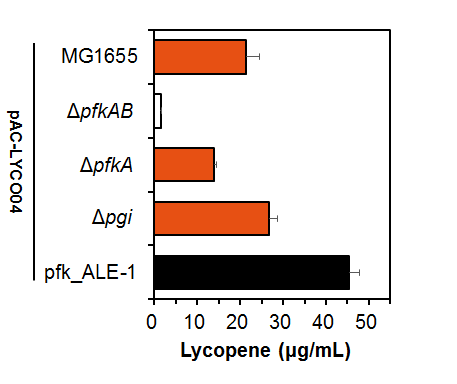


**b**


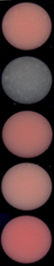

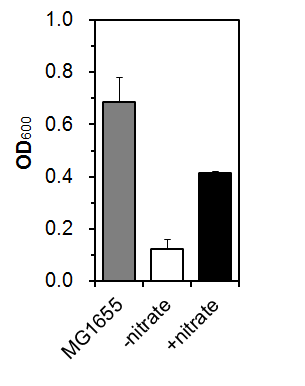


**c**


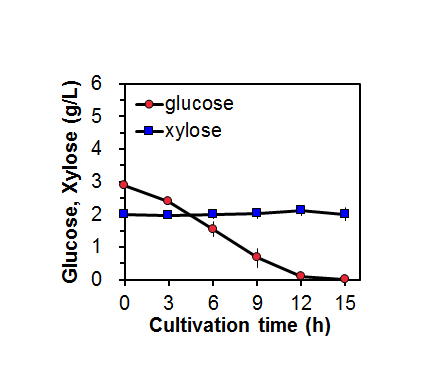


**d**

**e**


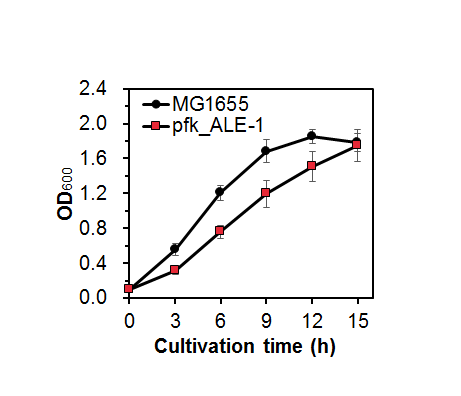


**f**

**Additional file 1 Figure S7. Diverse phenotypic characteristics of pfk_ALE-1.** (a, b) Lycopene production was examined in ALE-1 and control strains all harboring the pAC-LYCO04 plasmid. (a) Lycopene titer of *E. coli* strains after 24 h of cultivation were compared. (b) Color of lycopene-producing strains after 24 h of cultivation. Two millilitres of lycopene-producing cells were harvested after 24 h of cultivation and re-suspended in 200 µL of distilled water. After transfer to 96-well plates, images of cells were taken using a 96-well microplate reader. (c) When nitrate was used as the final electron acceptor under anoxic conditions, OD_600_ of ALE-1 and MG1655 were measured. For OD_600_ of MG1655 with nitrate addition, there was no significant difference caused by nitrate addition (data not shown). (d-f) Residual sugar concentration and cell growth of ALE-1 and MG1655 strains when grown in M9 minimal media supplemented with 3 g/L of glucose and 2 g/L of xylose. Residual glucose and xylose concentration of (d) MG1655 and (e) ALE-1 strain. (f) The cell growth of both strains is presented.

**
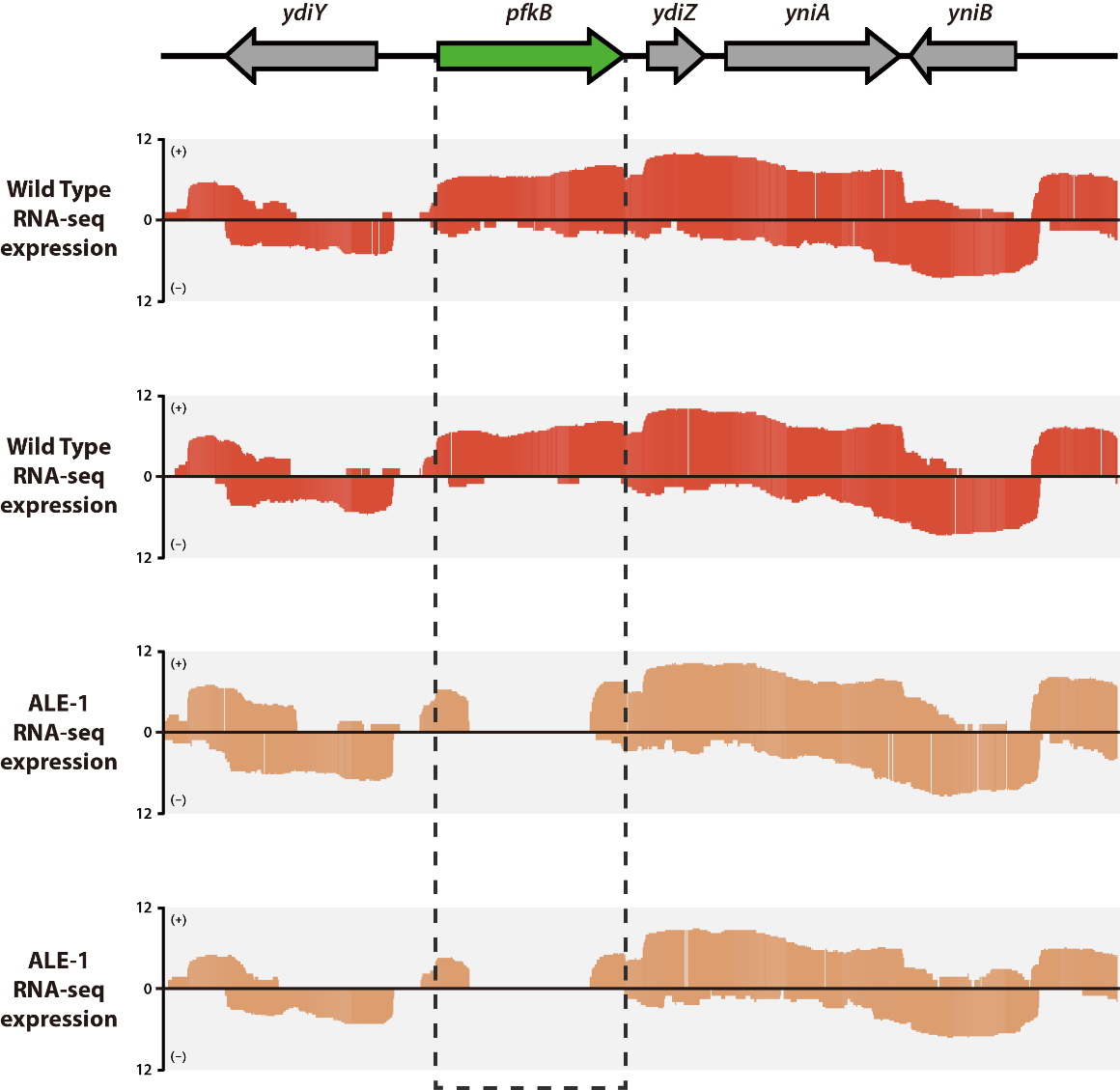
**

**Additional file 1 Figure S8. Unexpected expression level of the *pfkB* gene of ALE-1.** In the process of deletion of the *pfkB* gene, nucleotides of 157bp from the 5' end and 177bp from the 3' end were detected. Using bowtie software, the sequence reads were mapped onto the wild-type reference genome (NC_000913.2) with the maximum insert size option of 500bp. Remained coding sequence of the *pfkB* gene affected the expression level of the *pfkB* gene of ALE-1.

**Additional file 1 Tables**

**Additional file 1 Table S1. Strains and plasmid list**

| **Strains/plasmids** | **Genotype and description** | **Source** |
| --- | --- | --- |
| **Strains** |  |  |
| MG1655 | *E*. *coli* K-12 F^−^ λ^−^ *ilvG^−^ rfb-50 rph-1* | Lab stock |
| Δ*pfkA* | MG1655 Δ*pfkA*::FRT | This study |
| Δ*pgi* | MG1655 Δ*pgi*::FRT | This study |
| Δ*pfkAB* | Δ*pfkA* Δ*pfkB*::FRT | This study |
| pfk*_*ALE-1 | Δ*pfkAB* adapted in glucose minimal media | This study |
| pfk*_*ALE-2 | Δ*pfkAB* adapted in glucose minimal media | This study |
| pfk*_*ALE-3 | Δ*pfkAB* adapted in glucose minimal media | This study |
| pfk*_*ALE-4 | Δ*pfkAB* adapted in glucose minimal media | This study |
| pfk*_*ALE-5 | Δ*pfkAB* adapted in glucose minimal media | This study |
| Δ*pfkAB* *gntR*^c422a^ | Δ*pfkAB* Δ*gntR*::*gntR*^c422a^ (A141E) | This study |
| Δ*pfkAB* *galR*^g812a^ | Δ*pfkAB* Δ*galR*::*galR*^g812a^ (G271D) | This study |
| Δ*pfkAB* Δ(*ugd*-*gnd*-*wbbL*_*2*) | Δ*pfkAB* Δ(*ugd-gnd-wbbL-2*)::FRT | This study |
| Δ*pfkAB* Δ*ptsG* | Δ*pfkAB* Δ*ptsG*::FRT | This study |
| Δ*pfkAB* *crp*^g253t^ | Δ*pfkAB* Δ*crp*::*crp*^g253t^ (A85S) | This study |
| Δ*pfkAB* *talB*^c740t^ | Δ*pfkAB* Δ*talB*::*talB*^c740t^ (A247V) | This study |
| Δ*pfkAB* *gntR*^c422a^ *galR*^g812a^ Δ(*ugd*-*gnd*-*wbbL*_*2*) Δ*ptsG crp*^g253t^ *talB*^c740t^ | Δ*pfkAB* *gntR*^c422a^ Δ*galR*::*galR*^g812a^ Δ(*ugd-gnd-wbbL-2*)::FRT Δ*ptsG*::FRT Δ*crp*::*crp*^g253t^ Δ*talB*::*talB*^c740t^ | This study |
| ALE-1_Δ*gntR* | pfk*_*ALE-1 Δ*gntR*^c422a^::FRT | This study |
| ALE-1_*gntR*^wt^ | pfk*_*ALE-1 Δ*gntR*^c422a^::*gntR*^wt^ | This study |
| ALE-1_*gntR*^wt^ pEdd-Eda | ALE-1_*gntR*^wt^ harboring pEdd-Eda | This study |
| ALE-1_Δ*galR* | pfk*_*ALE-1 Δ*galR*^g812a^::FRT | This study |
| ALE-1_*galR*^wt^ | pfk*_*ALE-1 Δ*galR*^g812a^::*galR*^wt^ | This study |
| ALE-1_*galR*^wt^ pGalP | ALE-1_*galR*^wt^ harboring pGalP | This study |
| ALE-1_Δ*ptsG* | pfk*_*ALE-1 Δ*ptsG*::FRT | This study |
| ALE-1_Δ*edd* | pfk*_*ALE-1 Δ*edd*::FRT | This study |
| ALE-1_(*ugd*-*gnd*-*wbbL*)^wt^ | pfk*_*ALE-1 (*ugd-gnd-wbbL-2*)^+^ (2312bp deletion in *ugd-gnd-wbbL-2* was replaced by the wild type allele) | This study |
| ALE-1_(*ugd*-*gnd*-*wbbL*)^wt^ Δ*ugd* | ALE-1_(*ugd*-*gnd*-*wbbL*)^wt^ Δ*ugd*::FRT | This study |
| ALE-1_(*ugd*-*gnd*-*wbbL*)^wt^ Δ*gnd* | ALE-1_(*ugd*-*gnd*-*wbbL*)^wt^ Δ*gnd*::FRT | This study |
| ALE-1 pRFP | pfk*_*ALE-1 harboring pRFP | This study |
| ALE-1 pGnd | pfk*_*ALE-1 harboring pGnd | This study |
| ALE-1 pGnd-GntR | pfk*_*ALE-1 harboring pGnd-GntR | This study |
| MG1655 FabI^ts^ pMCR pACC | MG1655 Δ*fabI*::*fabI*^S241F^_FRT pMCR pACC | This study |
| Δ*pfkA* FabI^ts^ pMCR pACC | Δ*pfkA* Δ*fabI*::*fabI*^S241F^_FRT pMCR pACC | This study |
| Δ*pfkAB* FabI^ts^ pMCR pACC | Δ*pfkAB* Δ*fabI*::*fabI*^S241F^_FRT pMCR pACC | This study |
| Δ*pgi* FabI^ts^ pMCR pACC | Δ*pgi* Δ*fabI*::*fabI*^S241F^_FRT pMCR pACC | This study |
| pfk_ALE-1 FabI^ts^ pMCR pACC | pfk_ALE-1 Δ*fabI*::*fabI*^S241F^_FRT pMCR pACC | This study |
| ALE-1 pGnd FabI^ts^ pMCR pACC | pfk_ALE-1 Δ*fabI*::*fabI*^S241F^_FRT pGnd pMCR pACC | This study |
| ALE-1 pGnd-GntR FabI^ts^ pMCR pACC | pfk_ALE-1 Δ*fabI*::*fabI*^S241F^_FRT pGnd-GntR pMCR pACC | This study |
| MG-LacZ40a | MG1655 Δ*lacZ*_838-879_::P*_P3-BCD2_*-tetA | This study |
| MG1655 pAC-LYCO04 | MG1655 harboring pAC-LYCO04 | This study |
| Δ*pfkAB* pAC-LYCO04 | Δ*pfkAB* harboring pAC-LYCO04 | This study |
| ∆*pfkA* pAC-LYCO04 | ∆*pfkA* harboring pAC-LYCO04 | This study |
| ∆*pgi* pAC-LYCO04 | ∆*pgi* harboring pAC-LYCO04 | This study |
| pfk_ALE-1 pAC-LYCO04 | pfk_ALE-1 harboring pAC-LYCO04 | This study |
| **Plasmids** |  |  |
| pBbA6c-*rfp* | p15A *ori*, carrying P _LlacO-1_ promoter and *rfp*, Cm^R^ | [2] |
| pBbS6k-*rfp* | SC101 *ori*, carrying P_LlacO-1_ promoter and *rfp*, Kan^R^ | [2] |
| pBbE2k-*rfp* | ColE1 *ori*, carrying P_tetO_ promoter and *rfp*, Kan^R^ | [2] |
| pEdd-Eda | pBbA6c with Δ*rfp*::*edd-eda* (from MG1655), Cm^R^ | This study |
| pGalP | pBbS6k with Δ*rfp*::*galP* (from MG1655), Kan^R^ | This study |
| pRFP | pBbS5a-*rfp*; SC101 *ori*, carrying P_lacUV5_ promoter and *rfp*, Amp^R^ | [2] |
| pGnd | pRFP with Δ*rfp*::*gnd* (from MG1655), Amp^R^ | This study |
| pGnd-GntR | pRFP with Δ*rfp*::*gnd-gntR* (from MG1655), Amp^R^ | This study |
| pSIM5 | SC101-ts *ori*, carrying heat-shock inducible λ-RED recombinase system, Cm^R^ | [3] |
| pKD13 | Template for kanamycin cassette flanked by FRT sites, Km^R^ | [4] |
| pCP20 | SC101-ts *ori*, carrying constitutively expressed yeast FLP recombinase, Am^R^, Cm^R^ | [5] |
| pCDF-*mcrC**-*mcrN* | pCDF-*mcrC**(N391V/K557W/S565N) –*mcrN* | [6] |
| pMCR | pBbE2k-*mcrC**(N391V/K557W/S565N) –*mcrN* | This study |
| pBbA2k-*accDABC* | pBbA2k-*rfp* with Δ*rfp*::*accD*, *accA*, *accB* and *accC*, Km^R^ | [7] |
| pACC | pBbA6c-*rfp* with Δ*rfp*::*accD*, *accA*, *accB* and *accC*, Km^R^ | This study |
| pAC-LYC04 | p15A *ori*, carrying *ipiHp1* gene under P_trc_ promoter and constitutively expressed *crtE*, *crtB* and *crtI* gene for lycopene synthesis, Cm^R^ | [8] |
| pHexR-*gfp* | ColE1 *ori*, carrying *hexR* gene under P_lacIq_ promoter and *gfp* gene under P_LhexR_ promoter, Kan^R^ | This study |

**Additional file 1 Table S2. Primer List**

| **Primer** | **Sequence (5′-3′)** |
| --- | --- |
| *pfkA*-del-FP | GTAAAGGAATCTGCCTTTTTCCGAAATCATTAATACAGTTTTTTCGCGCAGTGTAGGCTGGAGCTGCTTCG |
| *pfkA*-del-RP | CTTCCGGCAACAGATTTCATTTTGCATTCCAAAGTTCAGAGGTAGTCATGATTCCGGGGATCCGTCGACC |
| *pfkA*-seq-FP | ATTATCCATCAGGACCCCTGTTCCG |
| *pfkA*-seq-RP | CGGAAGAAATCCGCCTCATATTGCT |
| *pfkB*-del-FP | ACTTTCCGCTGATTCGGTGCCAGACTGAAATCAGCCTATAGGAGGAAATGGTGTAGGCTGGAGCTGCTTC |
| *pfkB*-del-RP | ATTCCCCCAATGCTGGGGGAATGTTTTTGTTAGCGGGAAAGGTAAGCGTAATTCCGGGGATCCGTCGACC |
| *pfkB*-seq-FP | TCGAAAGGTTTTTTAGCGCTGGCA |
| *pfkB*-seq-RP | TGGGTAAGCCCCGAAACCAG |
| *gntR*-H1-P1 | GTCCGTGTTAAACTAAGAGAATCTATCTCTTTTGTACCTTCAGGACGATGGTGTAGGCTGGAGCTGCTTC |
| *gntR*-H2-P4 | CCATGCCTTAAGTGTATAAGTGTGAGCTACTTCAAATTTGTGGGCTTAAAATTCCGGGGATCCGTCGACC |
| *gntR*-seq-FP | GCCTTTTTGCAGGCTTCCTC |
| *gntR*-seq-RP | CCCGATACGCCCATCAAGAC |
| *galR*-tetA-P1 | CGTTCTCAATGATAATGGTATTGATGTACCGGGTGAGATTTCGTTAATTGGTGTAGGCTGGAGCTGCTTCG |
| *galR*-H2-P4 | CAGACCATCGAAGAATTACTGGCGCTGGAATTGCTTTAACTGCGGTTAGTATTCCGGGGATCCGTCGACC |
| *galR*-FP | TAATGAAACAAATGCCCGGT |
| *galR*-RP | TGCCGGAAGTGAAGGCAGGT |
| *galR*-seq-FP | GGCTCACATTCCCACGATG |
| *galR*-seq-RP | GGGCGATGTCTTTACCCAG |
| *ptsG*-H1-P1 | CACGCGTGAGAACGTAAAAAAAGCACCCATACTCAGGAGCACTCTCAATTGTGTAGGCTGGAGCTGCTTC |
| *ptsG*-H2-P4 | GTAAAAAAGGCAGCCATCTGGCTGCCTTAGTCTCCCCAACGTCTTACGGAATTCCGGGGATCCGTCGACC |
| *ptsG*-seq-FP | ATAACTTCGCCCGTCTGTTT |
| *ptsG*-seq-RP | ATGTTCACCAGCTATCGGTT |
| *ugd*-*gnd*-*wbbL*-del-FP | TAATCAGCATCCCGGTAGGCTTCATTTTTATCTAATGTGGCATTAAAGTGATTCCGGGGATCCGTCGACC |
| *ugd*-*gnd*-*wbbL*-del-RP | GAAATGACTGAGTCAGCCGAGAAGAATTTCCCCGCTTATTCGCACCTTCCGTGTAGGCTGGAGCTGCTTCG |
| *ugd*-*gnd*-*wbbL*-FP | TAATCAGCATCCCGGTAGGC |
| *ugd*-*gnd*-*wbbL*-RP | GAAATGACTGAGTCAGCCGAG |
| *wbbL*-2-ins-FP | TTATTAAAGTATAAATAGCTTATCCATGCTTATATGCTTACGGCTTTATAGTGTAGGCTGGAGCTGCTTC |
| *wbbL*-2-ins-RP | TTTTATCAAATCGCAACTTTGATCGAATTTCATCAGTTTTTCACCCGTAAATTCCGGGGATCCGTCGACC |
| *wbbL*-2-kanR-zzB-ins-FP | GTGTATACCTTATCTGCCACATCCTTAAGCTCTTCTGCCATTCGGTTAGAGATAATGACGTCGGTTCATGCTCTCAGAATTAACTTAAC |
| *ugd*-seq-RP | CATCATTACCCTAACTGACGG |
| *crp*-H1-P4 | GGCGTTATCTGGCTCTGGAGAAAGCTTATAACAGAGGATAACCGCGCATGATTCCGGGGATCCGTCGACC |
| *crp*-H2-P1 | CTACCAGGTAACGCGCCACTCCGACGGGATTAACGAGTGCCGTAAACGACGTGTAGGCTGGAGCTGCTTCG |
| *crp*-FP | TTGATGTACTGCATGTATGC |
| *crp*-RP | ATGGCGCGCTACCAGGTAAC |
| *crp*-seq-FP | ATAGCCCCTTCCCAGGTAG |
| *crp*-seq-RP | CAGGAACGAGGGAGAAGAG |
| *talB*-H1-P1 | AGACCGGTTACATCCCCCTAACAAGCTGTTTAAAGAGAAATACTATCATGGTGTAGGCTGGAGCTGCTTC |
| *talB*-H2-P4 | CTTCAGAAGAGGTAGCGTGACCGACTTCCCGGTCACGCTAAGAATGATTACAATTCCGGGGATCCGTCGACC |
| *talB*-FP | AAAGCAAAACGCCTGATCAA |
| *talB*-RP | GCGCAGGGATGCCTTTATCC |
| *talB*-seq-FP | CTGGCGATAACCGTCTTGTC |
| *talB*-seq-RP | CCTGCTCATCGGGGATTAAG |
| *Eco*RI-RBS-*edd*Ec-FP | CCCCCGAATTCTGACAACTCAATTTCAGGAGCCTTTATGAATCCACAACTACTGCGCGTAACAAATCGAATCATTG |
| *Bam*HI-*eda*Ec-RP | CCCGGATCCTTACAGCTTAGCGCCTTCTAC |
| *Bgl*II-RBS-*galP*Ec-FP | CCAGATCTAAAAATAACCATATTGGAGGGCATC |
| *Xho*I-*galP*Ec-RP | CCCCCTCGAGTTAATCGTGAGCGCCTATTTC |
| *gnd*-H1-P1 | AGGCCGCGAGCATTCAGCGCGGTGATCACACCTGACAGGAGTATGTAATGGTGTAGGCTGGAGCTGCTTC |
| *gnd*-H2-P4 | GTGCAATATACGCCGGGCCTCAATTTTATTGTTGGTTAAATCAGATTAATATTCCGGGGATCCGTCGACC |
| *gnd*-seq-FP-2 | GATTTGATGACCATAACCGC |
| *gnd*-seq-RP-2 | GTCAGTGGGAGAGATCTCAC |
| *Eco*RI-*Bgl*II-*gnd*-FP | CCCCCCGAATTCAAAAGATCTTGATCACACCTGACAGGAGT |
| *Xho*I-*Bam*HI-*gnd*-RP | GGGGGGCTCGAGTTTGGATCCTTAATCCAGCCATTCGGTATGG |
| *Eco*RI-*BglI*I-*gntR*-FP | CCCCCCGAATTCAAAAGATCTCTGGACGGAAGTCCAGGCCATAAACATGAAAAAGAAAAGACCCGTACTTC |
| *Xho*I-*Bam*HI-*gntR*-RP | GGGGGGCTCGAGTTTGGATCCTTAAATAGATCCGCCCGGTG |
| *fabI*(S241F)-km-SOE-P1 | TGTGGGTAACTCTGCGGCATTCCTGTGCTCCGATCTCTCTGCCGGTATCTTCGGTGAAGTGGTCCACGTTGACGGCGGTTTCAGCATTGCTGCAATGAACG |
| *fabI*(S241F)-km-SOE-P2 | GGTCGACGGATCCCCGGAATTTATTTCAGTTCGAGTTCGTTCATT |
| *fabI*(S241F)-km-SOE-P3 | AATGAACGAACTCGAACTGAAATAAATTCCGGGGATCCGTCGACC |
| *fabI*(S241F)-km-SOE-P4 | AACAGAGATAACGGGCGGCAGAACGCCGCCCATCTTTACCAACAGAACGAGTGTAGGCTGGAGCTGCTTC |
| (Km)P4-*fabI*-HR | TTAATTCTCATGTTTGACAGCCTGCTCCGGTCGGACCTGG |
| *fabI*-seqRP | ACCTTTGCGGCTTCCGGGAC |

**Additional file 1 Materials and Methods**

**Cell culture**

For anoxic growth, a single colony from an agar plate was used to inoculate 5 mL of LB medium. After growing for 9 h, cells were 1:10 diluted into 50 mL of M9 minimal media supplemented with 2 mM MgSO_4_, 0.1 mM CaCl_2_, and 5 g/L glucose in 250-mL shaking flasks. Overnight-grown cells were transferred to 30 mL of fresh M9 minimal media in 150-mL serum bottles. The initial OD_600_ was adjusted to approximately 0.2. Oxygen in the M9 minimal medium was removed by flushing with nitrogen gas and sealing. If required, 50 mM of sodium nitrate was added. During the entire process, the cells were grown at 37°C and 200 rpm. OD_600_ values was measured using a Libra S22 spectrophotometer.

For lycopene production, a single colony from an agar plate was used to inoculate 5 mL of LB medium supplemented with appropriate antibiotics at 37°C and 200 rpm. Overnight-grown cells were 1:10 transferred into 50 mL of LB medium supplemented with appropriate antibiotics and grown for 9 h. Thereafter, the pre-culture was 1:10 diluted into 50 mL of M9 medium supplemented with 2 mM MgSO_4_, 0.1 mM CaCl_2_, 4 g/L glucose and 100 mM potassium phosphate buffer (pH 7.0) with appropriate antibiotics. At the beginning of culture, 0.1 mM of IPTG was added to induce the expression of enzymes involved in lycopene synthesis.

**Quantitative RT-PCR**

For quantitative RT-PCR, cDNA was synthesized by employing 1 μg of total RNA using iScript cDNA Synthesis Kit (Bio-Rad, USA). A quantitative RT-PCR was performed using the StepOne Real-Time PCR system (Applied Biosystems, USA) based on the SYBR green method. The relative mRNA expression level was calculated using the ΔΔCt method [9]. The *rpoD* gene (housekeeping sigma factor 70) was used as a reference.

**Lycopene extraction and quantification**

Lycopene was extracted following a previously described method [8]. Briefly, cells were harvested by centrifuging 2 mL of cell culture for 1 min at 13,200 × *g*. After addition of 1 mL of acetone, lycopene was extract by vigorous vortex followed by heating for 15 min at 55°C. A 500-µL aliquot of the supernatant acquired after centrifuging for 3 min at 13,200 × *g* was mixed with an equal volume of distilled water. Absorbance was measured at 474 nm (A_454_) using a Libra S22 spectrophotometer and the concentration of lycopene was estimated by comparing with the A_454_ values of a standard lycopene solution.

**References**

1. Sauer U, Lasko DR, Fiaux J, Hochuli M, Glaser R, Szyperski T, et al. Metabolic flux ratio analysis of genetic and environmental modulations of *Escherichia coli* central carbon metabolism. *J Bacteriol*. 1999;**181(21)**:6679–88.
2. Lee TS, Krupa RA, Zhang F, Hajimorad M, Holtz WJ, Prasad N, et al. BglBrick vectors and datasheets: A synthetic biology platform for gene expression. *J Biol Eng*. 2011;**5**:15–7.
3. Datta S, Costantino N, Court DL. A set of recombineering plasmids for gram-negative bacteria. *Gene*. 2006;**379(1–2)**:109–15.
4. Datsenko KA, Wanner BL. One-step inactivation of chromosomal genes in *Escherichia coli* K-12 using PCR products. *Proc Natl Acad Sci U S A*. 2000;**97(12)**:6640–5.
5. Cherepanov PP, Wackernagel W. Gene disruption in *Escherichia coli*: TcR and KmR cassettes with the option of Flp-catalyzed excision of the antibiotic-resistance determinant. *Gene*. 1995;**158(1)**:9–14.
6. Liu C, Ding Y, Zhang R, Liu H, Xian M, Zhao G. Functional balance between enzymes in malonyl-CoA pathway for 3-hydroxypropionate biosynthesis. *Metab Eng*. 2016;**34**:104–11.
7. Shin KS, Lee SK. Introduction of an acetyl-CoA carboxylation bypass into *Escherichia coli* for enhanced free fatty acid production. *Bioresour Technol*. 2017;**245**:1627–33.
8. Kim SW, Keasling JD. Metabolic engineering of the nonmevalonate isopentenyl diphosphate synthesis pathway in *Escherichia coli* enhances lycopene production. *Biotechnol Bioeng*. 2001;**72(4)**:408–15.
9. Yim SH, Kim TM, Hu HJ, Kim JH, Kim BJ, Lee JY, et al. Copy number variations in East-Asian population and their evolutionary and functional implications. *Hum Mol Genet.* 2010;**19(6)**:1001–8.
